# Phosphorescent Pd^II^–Pd^II^ Emitter‐Based Red OLEDs with an EQE_max_ of 20.52%

**DOI:** 10.1002/advs.202404621

**Published:** 2024-07-19

**Authors:** Lige Qiao, Xiangjun Kong, Kechun Li, Lequn Yuan, Yunjun Shen, Yuzhen Zhang, Liang Zhou

**Affiliations:** ^1^ Key Laboratory of Chemistry and Engineering of Forest Products State Ethnic Affairs Commission Guangxi Key Laboratory of Chemistry and Engineering of Forest Products Guangxi Collaborative Innovation Center for Chemistry and Engineering of Forest Products School of Chemistry and Chemical Engineering Guangxi Minzu University Nanning Guangxi 530006 China; ^2^ State Key Laboratory of Rare Earth Resource Utilization Changchun Institute of Applied Chemistry Chinese Academy of Sciences Changchun 130022 P. R. China

**Keywords:** dinuclear palladium complexes, organic light‐emitting diode, phosphorescence, polymethylmethacrylate

## Abstract

Three dinuclear Pd(II) complexes (**1**, **2,** and **3**) with intense red phosphorescence at room temperature are here synthesized using strong ligand field strength compounds. All three complexes are characterized by nuclear magnetic resonance, high‐resolution mass spectrometry, and elemental analyses. Complexes **2** and **3** are characterized by single‐crystal X‐ray diffraction. The crystalline data of **2** and **3** reveal complex double‐layer structures, with Pd–Pd distances of 2.8690(9) Å and 2.8584(17) Å, respectively. Furthermore, complexes **1**, **2**, and **3** show phosphorescence at room temperature in their solid states at the wavelengths of 678, 601, and 672 nm, respectively. In addition, they show phosphorescence at 634, 635, and 582 nm, respectively, in the 2 wt.% (PMMA) films, and phosphorescence at 670, 675, and 589 nm, respectively, in the deoxygenated CH_2_Cl_2_ solutions. Among three complexes, complex **1** shows red emission at 634 nm with phosphorescent quantum yield *Ф* = 67% in the 2 wt.% PMMA film. Furthermore, complex **1**‐based organic light‐emitting diode is fabricated using a vapor‐phase deposition process, and their maximum external quantum efficiency reaches 20.52%, which is the highest percentage obtained by using the dinuclear Pd(II) complex triplet emitters with the CIE coordinates of (0.62, 0.38).

## Graphical abstract

Red emitters of Pd^II^–Pd^II^ are prepared with the highest quantum yield of 67% and their organic light‐emitting diode (OLED) maximum external quantum efficiency of 20.52% for the first time. These excellent performances indicate Pd^II^–Pd^II^ emitters’ great particle value in the OLED industry.

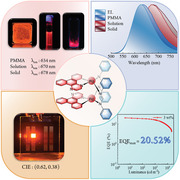

## 1 Introduction

With the development of organic light‐emitting diodes (OLEDs) in both academia and industry, the demand for highly efficient emitters has promoted the research in light‐emitting materials. The first‐generation emitters are pure organic dyes can collect as much as 25% of the singlet excitons in the electroluminescence process.^[^ ^1^, ^2^, ^3^, ^4^, ^5^ ^]^ To increase the device efficiencies by collecting the rest (i.e., 75%) of the triplet excitons, second‐generation emitters have been developed by using heavy atoms (such as Ir(III), Pt(II), and Au(III)) in the formation of phosphorescent transition metal complexes.^[^ ^6^, ^7^, ^8^, ^9^, ^10^, ^11^, ^12^, ^13^, ^14^ ^]^ Furthermore, the third‐generation emitters are thermally activated delayed fluorescence (TADF) materials with small energy gaps (Δ*E*_ST_) between the lowest‐lying singlet state S_1_ and the lowest‐lying triplet state T_1_. In addition, they consist of both pure organic compounds^[^ ^15^, ^16^, ^17^ ^]^ and transition metal complexes containing Cu(I),^[^ ^18^, ^19^, ^20^ ^]^ Pd(II),^[^ ^21^, ^22^ ^]^ and Au(III).^[^ ^23^ ^]^ However, the emitting materials used in industry are still dominated by phosphorescent transition metal complexes containing Ir(III) and Pt(II) elements.

Among these transition metal emitters, the Ir(III),^[^ ^24^, ^25^, ^26^, ^27^ ^]^ Pt(II),^[^ ^28^, ^29^ ^]^ Pt(III),^[^ ^30^ ^]^ and Au(III)^[^ ^31^ ^]^ complexes have been extensively explored. Since the first report on the green emitter *fac*‐Ir(ppy)_3_ by Watts et al.,^[^ ^32^ ^]^ many Ir(III) complexes with excellent OLED performances have been prepared by Thompson et al.^[^ ^33^, ^34^, ^35^, ^36^, ^37^ ^]^ In comparison with the Ir(III) triplet emitters, many Pt(II) complexes have also been synthesized by Che et al.^[^ ^38^, ^39^, ^40^, ^41^, ^42^, ^43^, ^44^, ^45^, ^46^, ^47^, ^48^, ^49^ ^]^ Furthermore, the Au(III) emitters are the third most important light‐emitting materials, and Yam et al.^[^ ^50^ ^]^ has reported on Au(III)‐based OLED devices with an external quantum efficiency (EQE) of 21.6%. In addition to these three transition metals, Thompson et al.^[^ ^51^ ^]^ reported 2019 on Cu(I) emitters with photoluminescence quantum yield (PLQY) > 99%. Also, Che et al.^[^ ^52^ ^]^ reported 2016 on Pd(II) emitters with tetradentate ligands, and they presented an EQE_max_ of 16.5% for the constructed OLEDs. Furthermore, Li et al.^[^ ^53^ ^]^ reported 2021 on Pd(II) emitters with an EQE_max_ of 34.8% of the constructed OLEDs. These superior Pd(II) emitters were modified by tetradentate ligands with high ligand field strengths by Li et al.^[^ ^54^, ^55^, ^56^, ^57^, ^58^ ^]^ Dinuclear triplet emitters for d_8_ transition metal of Pt were explored with great OLED performance by Chou et al.,^[^ ^59^ ^]^ Ma et al.,^[^ ^60^ ^]^ Che et al.,^[^ ^61^ ^]^ and Li's group.^[^ ^29^ ^]^ However, binuclear Pd(II) emitters have also been studied,^[^ ^62^, ^63^, ^64^ ^]^ but these complexes were all nonemissive due to the lower ligand field d–d fission energy level of Pd_2_ 4dσ*−4dσ* orbit and the smaller spin–orbit coupling constant of Pd (1054 cm^−1^). Thus, the preparation of bright dinuclear Pd(II) triplet emitters is still a challenge for the current triplet emitter research.

Three butterfly Pd^II^–Pd^II^ complexes with red emissions and other exceptional photophysical properties have been constructed in the present study. Complex **1** was prepared by using the conjugate C^N ligand of 1,4‐diazanaphthalene and the clamping ligand of *N*,*N*'‐diphenylformamidine (dpmd). The same C^N ligand was used for the preparation of complex **2**, but with the clamping ligand of 3, 5‐di‐tert‐butyl‐1*H*‐pyrazole. Furthermore, complex **3** was prepared by using the C^N ligand of 9‐phenyl‐2‐(pyridin‐2‐yl)−9*H*‐carbazole and the clamping ligand of dpmd. The purpose with the choice of these specific ligands was to implement their red emissions in the dinuclear Pd(II) complexes. Moreover, the three complexes were fully characterized by nuclear magnetic resonance (NMR), high‐resolution mass spectrometry (HRMS), and elemental analyses. Complexes **2** and **3** were also characterized by single crystal X‐ray diffraction (XRD), and their structures were found to have the forms of butterflies with strong Pd–Pd interactions. In addition, complexes **1**, **2**, and **3** showed triplet emissions from yellow to red at room temperature (r.t.) in their solid states. The emission wavelengths were then 678, 601, and 672 nm, respectively. In addition, the emission wavelengths were 634 nm, 635 nm, and 582 nm, respectively, for the complexes in CH_2_Cl_2_ solutions and 670, 675, and 589 nm, respectively, for the complexes in the 2 wt.% PMMA films. The complex **1** in the 2 wt.% PMMA film showed the highest quantum efficiency of 67%, with a lifetime of 4.68 µs. In view of the excellent photophysical properties of complex **1**, vapor‐phase deposition was used in the preparation of **1**‐based OLEDs. One of these devices showed the maximum external quantum efficiency (EQE_max_) of 20.52% and the maximum luminance of 20444 cd m^−2^, with Commission Internationale de l'Eclairage (CIE) coordinates of (0.62, 0.38). This excellent performance of a **1**‐based OLED did not only prove that butterfly dinuclear Pd(II) complexes are emissive at r.t., but also showed that it was possible to prepare high‐quality OLED emitters with the Pd transition metal.

## 2 Result and Discussions

### 2.1 Syntheses and Crystal Structures

**Figure 1:**
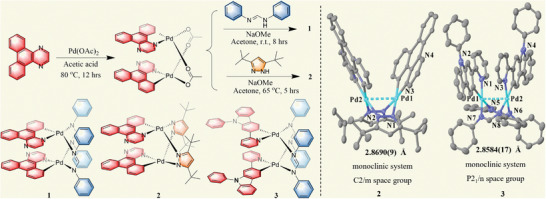
Synthetic routes, structures and geometrical structures of the Pd (II) complexes.

The synthesis processes of the different butterfly dinuclear Pd(II) complexes are illustrated in **Figure** **1**. The precursors in these processes (R1 and R2), with acetate as the clamping ligands, had been prepared in advance (Supporting information). The starting material R1 was treated with 2.5 equivalents of *N*,*N*'‐diphenylformamidine and 3.0 equivalents of sodium methylate in an acetone for 8 h at r.t. Complex **1** was, thereby, formed. It was, thereafter, purified by silica gel column chromatography, for which the CH_2_Cl_2_/hexane eluent was used. Complexes **2** and **3** were obtained by following the same procedure, except that the reaction temperature for complex **3** was 65 °C. All three complexes were, thereafter, characterized by NMR, HRMS, and elemental analyses. In addition, the chemical structures of complexes **2** and **3** were determined by single crystal XRD.

As the next step, the single crystals of complexes **2** and **3** were grown by slow evaporations of a complex **2**‐saturated solution of CH_2_Cl_2_/hexane and a complex **3**‐saturated solution of CH_2_Cl_2_/CH_3_OH, respectively. The XRD‐results revealed that both complexes **2** and **3** were crystallized in the monoclinic system, but with *C*2/*m* and *P*2_1_/*n* space groups, respectively. In these complexes, each of the Pd atoms had a square planar geometry. They were binding to C and N atoms in the C^N ligands of dibenzo[f,h]quinoxaline (**2**) and 9‐phenyl‐2‐(pyridin‐2‐yl)−9*H*‐carbazole (**3**), and to N atoms in the N^N ligand of 3,5‐di‐tert‐butyl‐1*H*‐pyrazole (**2**) and *N*,*N*'‐diphenylformamidine (**3**). For complex **2**, the Pd─C bond lengths were 2.009(6) Å and 2.028(6) Å, and the Pd─N bond lengths ranged from 2.009(6) to 2.090(5) Å. Similarly, for complex **3**, the Pd─C bond lengths were 1.997(8) Å and 2.034(7) Å, and the Pd─N bond lengths ranged from 1.951(7) to 2.124(7) Å (Tables S2 and S3, Supporting Information). Furthermore, the Pd–Pd distances of 2.869(9) Å and 2.858(17) Å (Figure 1) in complexes **2** and **3**, respectively, were equal to the sum of the two Pd radii which indicated the existence of Pd─Pd bonds.

### 2.2 Photophysical Properties and Electrochemistry

**Figure 2:**
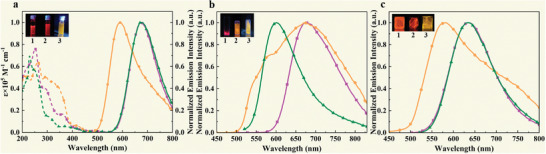
a) Absorption spectra (dashed lines) and normalized emission spectra (solid lines) of complexes **1**, **2**, and **3** (purple, green and orange, respectively) in deoxygenated CH_2_Cl_2_ solutions at r.t. Normalized emission spectra of complexes **1**, **2**, and **3** (purple, green and orange, respectively) in b) their solid states (i.e., powder) and c) 2 wt% PMMA films at r.t.

Moreover, the UV–Vis absorption spectra of the complexes **1**–**3** in CH_2_Cl_2_ solutions were measured at 298 K. The emission spectra, quantum yields, and lifetimes of these complexes were also measured in their solid states, in 2 wt% PMMA films, and in CH_2_Cl_2_ solutions. The absorption and emission spectra are presented in **Figure** **2**, and the photophysical data are presented in **Table** **1**. Complexes **1** and **3** exhibited strong absorption bands at 250 nm with a molar extinction coefficiency *ε* > 6.0 × 10^4^ M^−1^ cm^−1^. Also, complex **2** exhibited a strong absorption band at 230 nm with ε > 6.9 × 10^4^ M^−1^ cm^−1^. These high‐energy absorption bands were assigned to intraligand (IL) charge transfer within the C^N and N^N ligands. Furthermore, the moderate absorption bands between 300 and 380 nm were assigned to ligand‐to‐ligand charge transfer (LLCT), and the low‐energy absorption bands for wavelengths larger than 410 nm, with *ε* ˂ 6 × 10^3^ M^−1^ cm^−1^, were assigned to MMLCT from 4d_z_^2^σ* to π_C^N_^*^, or from 4d_z_^2^σ* to π_ppy_^*^. Moreover, complexes **1**, **2**, and **3** showed phosphorescence in N_2_ degassed CH_2_Cl_2_ solutions with the maximum wavelengths of 670, 675, and 589 nm, respectively. Complexes **1** and **2**, with different N^N ligands, showed almost the same emission, which indicated that the emissions of these two complexes were dominated by an MMLCT from 4d_z_^2^σ* to π_C^N_*. Also, the emission spectra of the three complexes in their solid states are presented in Figure 2b. The maximum wavelengths of these emission spectra were 678 nm (**1**), 601 nm (**2**), and 672 nm (**3**). Complex **3** exhibited broad emission at solid state is properly because of the π–π interaction between two adjacent molecules (Figure S11, Supporting information). The pendent phenyl rings in complex **3** show intensive π–π interaction at solid state and this interaction results in the emission band at 650–750 nm. This band accompanied with the band at 570 nm having the nature of MMLCT exhibited the broad spectra for complex **3** at solid state. However, with the breakdown of the π–π interaction between two pendent phenyl rings in diluted solution and PMMA film, the intensity of the emission band at 650–750 nm decreased to shoulder peaks. The spectra of complexes **1** and **3** were red shifted by 8 and 83 nm, respectively, in comparison with their spectra in the CH_2_Cl_2_ solutions. Also, they were red shifted by 44 nm and 90 nm, respectively, in comparison with their spectra in the 2 wt.% PMMA films. On the contrary, the spectrum of complex **2** in the solid‐state was blue shifted by 74 and 34 nm in comparison with the spectra for the CH_2_Cl_2_ solution and PMMA film, respectively. This blue‐shift is abnormal for Pt and Pd complexes. In the case of complex **2**, it can be ascribed to the Pd─Pd bond length extension induced blue shift at solid state (Figure S9, Supporting Information). With the crystallization of complex **2** at solid state, the ligands of 1,4‐diazanaphthalene staggered with each other and formed π–π interaction. This π–π interaction could induce red‐shift for Pd and Pt complexes for most of the complexes. However, the formation of π–π interaction and the insertion of additional ligand 1,4‐diazanaphthalene into the space between two 1,4‐diazanaphthalene ligands of one single molecule extended the Pd–Pd bond length. Based on our experiment data, the longer of the bond length for Pd–Pd, the stronger blue shift for binuclear Pd complexes. Additional experiments were performed to verify this hypothesis (Figures S17–S20, Supporting Information). With the disappear of π–π interaction in solution and doped PMMA films, the emission of complex **2** show red shift. But for the thin film prepared with complex **2** in 100% concentration, it exhibited pretty similar spectrum with its spectrum at solid state. Furthermore, a comparison of the emission wavelengths with the excitation wavelengths in the various spectra revealed that the complexes showed relatively large Stoke shifts (>100 nm) and long excited state lifetimes (in the range of microseconds). This indicated that the intrinsic radiative decays of the complexes originated from their triplet excited states. The emission wavelengths for complexes **1**, **2**, and **3** were 634, 635, and 582 nm, respectively, in the 2 wt.% PMMA films. Thus, complexes **1** and **2** had almost identical emission wavelengths in the 2 wt.% PMMA films. Complex **1** was blue shifted by 44 nm and 36 nm compared with the wavelengths in its solid state and in the CH_2_Cl_2_ solution, respectively. In addition, complex **3** was blue shifted by 90 and 7 nm, respectively. Also, complex **2** was red‐shifted by 34 nm compared to its solid state, and it was blue shifted by 40 nm compared to the situation with **2** in the CH_2_Cl_2_ solution. Furthermore, the lifetimes for the three complexes were in the microsecond range of 2.05–14.86 µs. The quantum yields for the three complexes in their different states were also measured and are summarized in Table 1. The quantum yields for complexes **1**, **2**, and **3** in the 2 wt.% PMMA films were 67%, 20%, and 1.3%, respectively. The low PLQY of complex **3** could be ascribed to the freely rotation of pendent phenyl ring in 9‐phenyl‐2‐(pyridin‐2‐yl)−9*H*‐carbazole in solution and PMMA film. However, the low PLQY at solid state could be ascribed to the formation of π–π interaction (Figure S11, Supporting Information) between two phenyl rings from adjacent molecules which disturbed the complex essential radiative decay of MMLCT at solid state. Moreover, the ligands flappy of carbazole plane and pyridine plane in 9‐phenyl‐2‐(pyridin‐2‐yl)−9*H*‐carbazole also populated the nonradiative decay process and decreased the PLQY for complex **3** (Figure S13, Supporting Information). This indicated that the quantum efficiencies were strongly related to the organic ligands, and the 1,4‐diazanaphthalene and *N*, *N*'‐diphenylformamidine compounds were found to be excellent ligands for the improvement of the quantum yields of the dinuclear Pd(II) complexes.

**Table 1:** Photophysical parameters and Electrochemical parameters for complexes.

Complex	Absorption^a)^	Medium (298 K)	Emission wavelength [λ nm^−1^]	*Ф*_PL_ ^d)^ [%]	*τ* [µs]	*k*_r_ ^e)^ (10^4^s^−1^)	*k*_nr_ ^e)^ (10^5^s^−1^)	*E*_ox_ [V]	*E*_HOMO_ [eV]^f)^	*E*_LUMO_ [eV]^g)^	*E*_g_ ^h)^ [eV]
1	251(76090), 360(16697), 410 (4608)	Solid	678	24	2.05	11.9	3.7	0.806/ 1.035	−5.24	−2.64	2.60
		PMMA^b)^	634	67	4.68	14.3	0.7				
		CH_2_Cl_2_ ^c)^	670	26	3.14	8.4	2.3				
2	229(69335), 368 (5533), 500 (1030)	Solid	601	15	2.89	5.0	3.0	0.846	−5.34	−2.88	2.46
		PMMA^b)^	635	20	4.47	4.5	1.8				
		CH_2_Cl_2_ ^c)^	675	16	2.39	6.6	3.5				
3	262(64083), 293(50363), 420 (5959)	Solid	672	4.0	5.28	0.8	1.8	0.604/ 1.022/ 1.178	−5.05	−2.10	2.95
		PMMA^b)^	582	1.3	14.86	0.1	0.7				
		CH_2_Cl_2_ ^c)^	589	1.5	2.18	0.7	4.5				

^a)^ Electronic absorption band maxima (*λ*_abs_) and molar absorption coefficients (log *ε*) in CH_2_Cl_2_ at room temperature;

^b)^ Recorded in 2 wt.% PMMA films;

^c)^ Recorded in N_2_‐degassed CH_2_Cl_2_ at 1 × 10^‐5^ mol L^‐1^;

^d)^ Phosphorescent quantum yield measured using an integrating light sphere;

^e)^ The radiative rate constant (*k*_r_) and nonradiative rate constant(*k*_nr_) were calculated from the quantum yield and lifetime using the following mathematical equations: *k*_r_ = *Ф*/τ and *k*_nr_ = (1 − *Ф*)/τ;

^f)^ HOMO energy levels that are calculated from CV data using ferrocene as an internal standard;

^g)^ LUMO energy levels that are calculated from CV data and the UV–Vis onset;

^h)^ Optical bandgaps from absorption spectra of complexes in degassed THF solutions, as determined by *E*_g_ = *hc*/*λ*_onset_.

Moreover, the cyclic voltammetry (CV) of the three complexes in THF solutions were measured by using ferricenium/ferrocene (Fc^+^/Fc) as a reference and n‐Bu_4_NPF_6_ as a supporting electrolyte. Complex **1** exhibited the first oxidation peak at 0.806 V and the second oxidation peak at 1.035 V. There was only one oxidation peak for complex **2**, which was observed at 0.846 V. Furthermore, the HOMO energy levels (*E*_HOMO_) for the three complexes were estimated by using the measured oxidation potential/optical band gaps (*E*_ox_/*E*_g_) values, and are presented in Table 1. Also, the corresponding LUMO energy levels (*E*_LUMO_) were calculated from the CV data and UV–Vis onset. The HOMO energy levels of complexes **1**, **2**, and **3** were −5.24, −5.34, and −5.05 eV respectively, and the LUMO energy levels were −2.64, −2.88, and −2.10 eV respectively.

### 2.3 Electroluminescence

**Figure 3:**
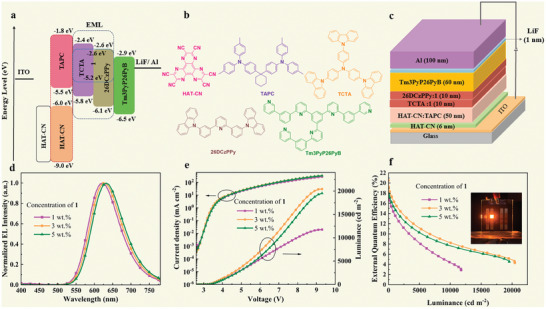
For a device with a double EML: a) Proposed energy‐level diagram. b) Molecular structure of each layer in the device. c) Device structure. d) Normalized electroluminescence (EL) spectra for three different concentrations of complex **1**. e) Current density−voltage−luminance (*J*−*V*−*L*) characteristics for three different concentrations of complex **1**. f) External quantum efficiency (EQE) characteristics for three different concentrations of complex **1**.

In the present study, complexes with good luminescence properties were selected for the preparations of OLEDs. Device structures with single‐ and double‐emitting layers (EMLs) were then designed with complex **1** as the luminescent material. The single‐EML structure was HAT‐CN (6 nm)/HAT‐CN (0.3 wt.%): TAPC (50 nm)/**1** (X wt.%):26DCzPPy (10 nm)/Tm3PyP26PyB (60 nm)/LiF (1 nm)/Al (100 nm). Also, the double‐EML structure was HAT‐CN (6 nm)/HAT‐CN (0.3 wt.%): TAPC (50 nm)/**1** (Y wt.%): TCTA (10 nm)/**1** (Y wt.%): 26DCzPPy (10 nm)/Tm3PyP26PyB (60 nm)/LiF (1 nm)/Al (100 nm). X and Y were set to 1, 3, and 5 in the different devices. The proposed energy level diagram for a device with a double‐EML structure is presented in **Figure** **3**a. In addition, the chemical structures of the organic materials that were used in the corresponding transporting and emitting layers are presented in Figure 3b. Among these structures, 1,4,5,8,9,11‐hexaazatriphenylene‐hexacarbonitrile (HAT‐CN) was used as a hole‐injection layer (HIL). Also, di‐[4‐(*N*,*N*‐ditolyl‐ amino)‐phenyl]cyclohexane (TAPC) was used as an electron‐blocking layer (EBL) and a hole‐transporting layer (HTL). Furthermore, 4,4′,4″‐tris(carbazol‐9‐yl)triphenylamine (TCTA) and 2,6‐bis(3(9*H*‐carbazol‐9‐yl)phenyl)pyridine) (26DCzPPy) were the two host materials. Complex **1** was used as the emitting material in the EML, and the host materials were doped with **1** at different concentrations (1%, 3%, or 5%). In addition, 1,3,5‐tri(6‐(3‐(pyridine‐3‐yl)phenyl)pyridine‐2yl) (Tm3PyP26PyB) functioned as both a hole‐blocking layer (HBL) and an electron transport layer (ETL). Finally, the LiF layer served as an electron injection layer (EIL) and Al was the cathode. The device performances are presented in Figure 3d–f, and the key data are summarized in **Table** **2**.

**Table 2:** Key performances of **1**‐based OLEDs with various doping concentrations of complex **1**.

Device	L [cd m^−2^]^a)^	*η*_c_ [cd A^−1^]^b)^	*η*_p_ [lm W^−1^]^c)^	EQE [%]^d)^	CIE*_x_*_,_ *_y_* ^e)^
1 wt%	11763	19.64	21.27	18.44	(0.61, 0.38)
3 wt%	20444	23.14	24.24	20.52	(0.62, 0.38)
5 wt%	19552	18.73	18.09	17.94	(0.63, 0.37)

^a)^ Maximum luminance (*L*);

^b)^ Maximum current efficiency (*η*_c_);

^c)^ Maximum power efficiency (*η*_p_);

^d)^ Maximum external quantum efficiency (EQE);

^e)^ Commission Internationale del'Eclairage (CIE*_x_*_,_ *_y_*) coordinates at 10 mA cm^−2^.

The performances of the OLEDs with single EMLs were not as excellent as those of the double‐EMLs, so the former type of OLEDs has not been further considered. However, their device characteristics and key data are presented in Figures S24–S26 and Table S4 (Supporting information), respectively. For the OLEDs with double‐EMLs, the electroluminescence (EL) spectra became red shifted for an increasing doping concentration of the light‐emitting complex **1** (Figure 3d). It was, thereby, assumed that the increase in doping concentration led to an increase in electron–hole recombination. As can be seen in Figure 3e. At the same voltage, the current density first increases and then decreases with the increase of complex **1** doping concentration. The reason may be that the doping ratio at a concentration of 3 wt% will balance the charge carriers in the device more efficiently, thereby being more efficient EMLs. At a doping concentration of 3 wt%, the device performance reached its highest level with a maximum luminance of 20444 cd m^−2^ and a maximum external quantum efficiency of 20.52%, with CIE coordinates of (0.62, 0.38).

## 3 Conclusion

In summary, by utilizing cyclometalated conjugated C^N ligands and clamping N^N ligands, this is the pioneer report on three butterfly dinuclear Pd(II) complexes with intense red or yellow phosphorescence at room temperature. Their photophysical properties have been analyzed, and complex **1** exhibited red emission at 634 nm with the highest quantum yield of 67% in 2 wt.% PMMA films. Moreover, **1**‐based OLEDs were fabricated with the maximum external quantum efficiency of 20.52%, with CIE coordinates of (0.62, 0.38). This study has not only provided a strategy for the preparation of phosphorescent Pd emitters, but it has also explored a new field of Pd emitters for applications in OLEDs.

## 4 Experimental Section

[2366852, 2310045, and 2284482 contain the supplementary crystallographic data for this paper. These data can be obtained free of charge from The Cambridge Crystallographic Data Centre via www.ccdc.cam.ac.uk/data_request/cif.]

## Conflict of Interest

The authors declare no conflict of interest.

## Supporting information

Supporting Information

## Data Availability

The data that support the findings of this study are available in the supplementary material of this article.
