# Supplementary material for: Phosphorescent PdII–PdII Emitter‐Based Red OLEDs with an EQEmax of 20.52%
Source: Adv Sci (Weinh). 2024 Jul 19;11(35):2404621. doi: 10.1002/advs.202404621 (PMC11425235; doi:10.1002/advs.202404621)
Supplement: Supplementary file 1 — Supporting Information [file ADVS-11-2404621-s001.docx]

Supporting Information

Phosphorescent Pd^II^-Pd^II^ Emitter-Based Red OLEDs with an EQE_max_ of 20.52 %

Lige Qiao ^#^ Xiangjun Kong ^#^ Kechun Li, Lequn Yuan, Yunjun Shen, Yuzhen Zhang,* and Liang Zhou*

Lige Qiao and Xiangjun Kong contributed equally to this work.

Contents

[1. General Information 1](#_Toc169346456)

[2. Experimental Section 2](#_Toc169346457)

[3. X-ray Crystallography 6](#_Toc169346458)

[4. Photophysical Properties 10](#_Toc169346459)

[5. Cyclic voltammetry data 12](#_Toc169346460)

[7. OLEDs fabrications 13](#_Toc169346461)

[5. NMR and HRMS 15](#_Toc169346462)

[8. References 20](#_Toc169346463)

# 1. General Information

**Chemicals**

If not specified, reagents and organic solvents are purchased directly and can be used without further purification.

**Characterization**

Thin-layer chromatography (TLC) was carried out using silica gel 60, F254 with a thickness of 0.25 mm. Column chromatography was performed on silica gel 60 (200-300 mesh).

NMR spectra were recorded on a Bruker AVANCE Ⅲ 400 spectrometer (^1^H NMR 400 MHz, ^13^C NMR 101 MHz) or 600 spectrometer (^1^H NMR 600 MHz, ^13^C NMR 151 MHz)at 25 °C. Data are reported as follows: Chemical shift in ppm, multiplicity (s = singlet, d = doublet, t = triplet, q = quartet, m = multiplet, dd = doublet of doublets, etc.), coupling constant J in Hz, integration, and (where applicable) interpretation.

High-resolution MS data were recorded using ThermoFisher Scientific (USA) equipped with an electrospray ionization source (ESI). Accurate mass determination was corrected by calibration using sodium trifluoroacetate clusters as a reference.

UV-vis absorption measurements were carried out on an Agilent’s Cary 100 UV−vis spectrophotometer. Emission spectrum, phosphorescence lifetime and quantum yield were measured directly using Edinburgh Instruments model FLS1000.

Cyclic voltammetry measurements were performed on the CHI 760E electrochemical workstation equipped with a glassy carbon working electrode (d= 2 mm), platinum wire counter electrode, Pt counter electrode, and Ag^+^/Ag reference electrode. ACS reagent grade solvents were used for the measurements.

All organic materials were obtained from commercial sources and were used without further purification. Pre-patterned indium tin oxide (ITO) coated glass (10 Ω sq^-1^) was used as an anode substrate and was carefully cleaned before use. Organic and metallic materials were grown in a vacuum chamber below 1.0 × 10^-5^ Pa, respectively. The evaporation rate of HAT-CN in the hole injection layer was controlled at 0.01 nm s^-1^, and that of TAPC in the hole transport layer was controlled at 0.05 nm s^-1^. When evaporating the luminescent layer, the luminescent material and the host material were evaporated simultaneously from different sources, and the evaporation rate of the host material was controlled at 0.05 nm s^-1^, and the evaporation rate of the luminescent material was adjusted according to the difference in doping concentration. The evaporation rate of Tm3PyP26PyB in the electron transport layer was controlled at 0.05 nm s^-1^. The evaporation rates of LiF and Al were controlled at 0.01 nm s^-1^ and 0.5 nm s^-1^, respectively. All the devices were characterized without encapsulation at room temperature. The current density-voltage-luminance (J-V-L) characteristics, EQE, CIE_x, y_, and EL spectra of the devices were measured using the the M6100 OLED IVL test system.

# 2. Experimental Section


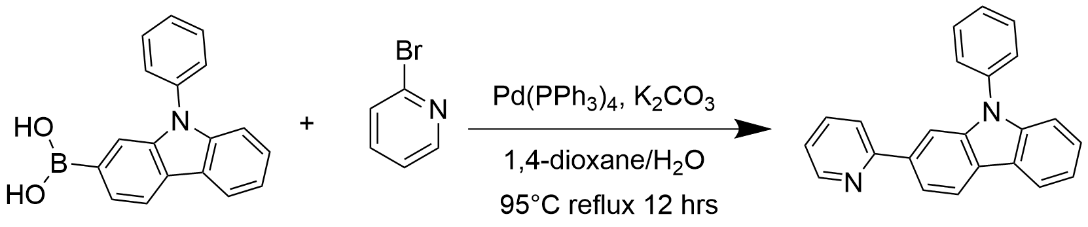


Figure S1. Synthesis route of 9-phenyl-2-(pyridin-2-yl)-9*H*-carbazole.

(9-Phenyl-9*H*-carbazol-2-yl)boronic acid (600 mg, 2.1 mmol), 2-bromopyridine (0.3 mL, 1.9 mmol), and potassium carbonate (788 mg, 5.7 mmol) were added to an oven-dried Schlenk tube, and 30 mL of 1,4-dioxane and 6 mL of deionized water were added in a nitrogen atmosphere. Tetrakis(triphenylphosphine)palladium (110 mg, 0.1 mmol) was then added in a nitrogen atmosphere, connected to a condenser tube and heated at 95 °C for 12 hours. After completion of the reaction, the solvent was removed under reduced pressure and the mixture was extracted with dichloromethane and washed three times with deionized water. The solution was then dried with magnesium sulfate and concentrated. 9-phenyl-2-(pyridin-2-yl)-9*H*-carbazole (481 mg, 79% yield) was purified by silica gel column chromatography^[1]^ .

**
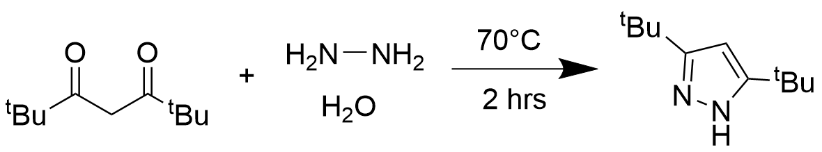
**

**Figure S2.** Synthesis route of 3,5-di-tert-butyl-1*H*-pyrazole.

3,5-di-tert-butyl-1*H*-pyrazole was synthesized following the procedure described in the literature^[2]^ . 2,2,6,6-tetramethyl-3,5-heptanedione (0.57 mL, 2.71 mmol) and hydrazine hydrate (0.14 mL, 2.71 mmol) were added to a 100 mL pressurized flask and stirred at 70°C for 2 hours. After completion of the reaction, the precipitated white solid was washed with methanol and dried. Finally the white crystalline product 3,5-di-tert-butyl-1*H*-pyrazole (400 mg, 82% yield) was obtained by recrystallization.


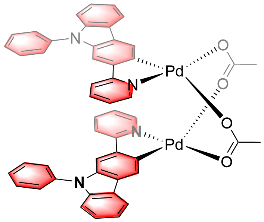


**Figure S3**. Structure of R2.

R2 was synthesized according to the procedure described in the literature^[3]^ . 9-Phenyl-2-(pyridin-2-yl)-9*H*-carbazole (500 mg, 1.56 mmol) and 40 ml of dichloromethane were added to a 100 ml round bottom flask. Palladium(II) acetate (350 mg, 1.56 mmol) was then added and stirred for 10 hours at room temperature. The solution is then concentrated and the solid residue is washed three times with ether until the ether is colorless. The complex is obtained as R2 (643 mg, 85% yield).

^1^H NMR (600 MHz, Methylene Chloride-*d_2_*) δ 8.03 (d, J = 7.7 Hz, 2H), 7.85 (dd, J = 5.6, 0.8 Hz, 2H), 7.62 (d, J = 8.1 Hz, 4H), 7.50 – 7.45 (m, 8H), 7.39 (dd, J = 6.9, 1.3 Hz, 2H), 7.34 (d, J = 8.1 Hz, 2H), 7.30 – 7.24 (m, 4H), 6.93 (d, J = 6.9 Hz, 4H), 6.04 (ddd, J = 7.1, 5.6, 1.6 Hz, 2H), 2.37 (s, 6H).^13^C NMR (151 MHz, Methylene Chloride-*d_2_*) δ 181.82, 164.61, 150.01, 142.04, 141.74, 141.55, 138.86, 138.08, 137.97, 137.12, 130.42, 129.96, 127.75, 127.22, 127.01, 126.61, 123.93, 123.05, 122.93, 120.99, 120.79, 120.29, 117.61, 109.92, 104.33, 25.25.


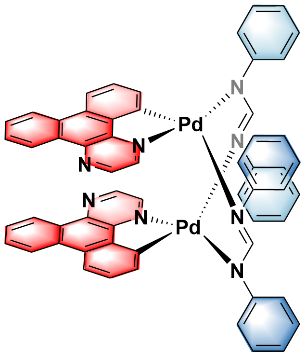
**Figure S4**. Structure of complex **1**.

Complexes **1** and **3** were obtained by dissolving the reactants in acetone and stirring. The complexes R1 (200 mg, 0.25 mmol) and *N*,*N*'-diphenylformamidine (149 mg, 0.76 mmol) were used according to the described procedure, followed by the addition of NaOMe (41 mg, 0.76 mmol) and stirring at room temperature. After completion of the reaction, the solution was concentrated, washed with methanol and water. The eluent with n-hexane: dichloromethane = 3:1 is used for purification through column chromatography. The initially purified solid complex is dissolved in dichloromethane, filtered and placed in a sample bottle, where one-fifth of the volume of n-hexane is added and slowly evaporated. When a quarter of the mixed solution is left, the remaining solution is removed and the solid is washed with a mixture of n-hexane and dichloromethane (n-hexane: dichloromethane = 5:1) to obtain complex **1** (142 mg, 53% yield).

^1^H NMR (400 MHz, Methylene Chloride-*d*_2_) δ 8.27 (d, *J* = 8.2 Hz, 2H), 8.22 (s, 2H), 7.91 (t, *J* = 7.2 Hz, 4H), 7.77 (d, *J* = 2.8 Hz, 2H), 7.69 – 7.64 (m, 6H), 7.49 (d, *J* = 7.3 Hz, 4H), 7.39 – 7.28 (m, 10H), 7.20 (d, *J* = 2.8 Hz, 2H), 7.16 – 7.11 (m, 4H), 7.06 (t, *J* = 7.4 Hz, 2H), 6.91 (t, *J* = 7.3 Hz, 2H). ^13^C NMR (101 MHz, Methylene Chloride-*d*_2_) δ 162.18, 157.78, 152.04, 151.66, 148.18, 142.77, 142.71, 141.57, 138.91, 133.29, 132.17, 131.19, 129.93, 129.72, 129.16, 129.14, 128.58, 127.71, 124.64, 124.39, 123.52, 122.91, 122.66, 122.40, 117.94.

HRMS (ESI): m/z Calcd. for C_58_H_40_N_8_Pd_2_, [M+H]^+^, 1063.1522; found, 1063.1527.

Elemental analysis: Calc. for C_58_H_40_N_8_Pd_2_: C, 65.05%; H, 3.86%; N, 10.46% found: C, 65.04%; H, 3.62%; N, 10.44%.

**
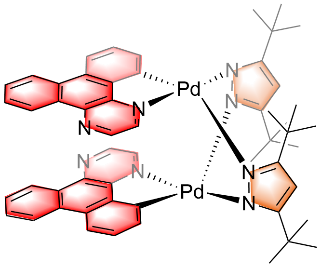
Figure S5**. Structure of complex **2**.

The synthesis steps of complex **2** is obtained by heating the reactant in acetone. Following the steps as described, the complex R1 (100 mg, 0.13 mmol), 3,5-di-tert-butyl-1*H*-pyrazole (69 mg, 0.38 mmol), and NaOMe (21 mg, 0.38 mmol) were heated to 65℃. After the reaction was completed, the solution was centrifuged and purified with methanol and water. The washed solid complex is dissolved in dichloromethane, filtered and placed in a sample bottle, where one-fifth of the volume of n-hexane is added and slowly evaporated. When a quarter of the mixed solution is left, the remaining solution is removed and the solid is washed with a mixture of n-hexane and dichloromethane (n-hexane: dichloromethane = 5:1) to obtain complex **2** (17.0 mg, 13% yield).

^1^H NMR (400 MHz, Chloroform-*d*) δ 8.52 (d, *J* = 9.4 Hz, 2H), 8.29 (d, *J* = 2.8 Hz, 2H), 7.93 (s, 1H), 7.91 (d, *J* = 2.9 Hz, 3H), 7.60 (d, *J* = 8.0 Hz, 2H), 7.45 – 7.37 (m, 4H), 7.00 (t, *J* = 7.7 Hz, 2H), 6.76 (d, *J* = 6.9 Hz, 2H), 6.13 (s, 2H), 1.63 (s, 18H), 1.56 (s, 18H). ^13^C NMR (101 MHz, Chloroform-*d*) δ 159.84, 159.16, 157.02, 149.03, 142.83, 142.76, 141.97, 138.34, 134.43, 132.12, 130.23, 129.73, 128.65, 128.24, 127.09, 124.44, 122.63, 117.36, 100.25, 32.58, 32.42, 32.37, 32.11, 31.74, 22.81, 14.27.

HRMS (ESI): m/z Calcd. for C_54_H_56_N_8_Pd_2_, [M+Na]^+^, 1053.2594; found, 1053.2600.

Elemental analysis: Calc. for C_54_H_56_N_8_Pd_2_: C, 62.43%; H, 5.53%; N, 10.79% found: C, 62.17%; H, 5.38%; N, 10.54%.


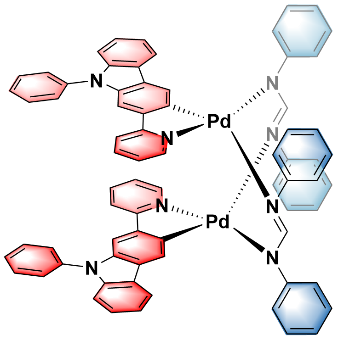


**Figure S6.** Structure of complex **3**.

Following similar steps to the preparation of complex **1**, complex R2 (200 mg, 0.206 mmol), *N*,*N*'-diphenylformamidine (121 mg, 0.619 mmol) and NaOMe (33.0 mg, 0.619 mmol) were used to obtain complex **3** (128 mg, 50% yield).

^1^H NMR (400 MHz, Methylene Chloride-*d*_2_) δ 8.23 (s, 2H), 7.95 (d, *J* = 7.8 Hz, 2H), 7.77 (s, 2H), 7.70 (d, *J* = 7.9 Hz, 4H), 7.63 (t, *J* = 7.7 Hz, 4H), 7.54 – 7.44 (m, 12H), 7.35 (d, *J* = 6.6 Hz, 4H), 7.28 (t, *J* = 7.8 Hz, 4H), 7.25 – 7.20 (m, 2H), 7.12 (t, *J* = 7.8 Hz, 4H), 7.04 – 6.94 (m, 6H), 6.88 (dt, *J* = 20.0, 7.3 Hz, 4H), 5.77 (t, *J* = 6.4 Hz, 2H). ^13^C NMR (101 MHz, Methylene Chloride-*d*_2_) δ 165.25, 162.17, 152.73, 152.30, 150.97, 147.37, 143.45, 141.70, 138.79, 138.29, 136.56, 130.38, 128.92, 127.58, 127.17, 126.27, 126.06, 124.71, 124.56, 123.42, 122.94, 122.58, 122.14, 120.85, 120.58, 120.17, 117.56, 109.80, 104.51.

HRMS (ESI): m/z Calcd. for C_72_H_52_N_8_Pd_2_, [M+H]^+^, 1243.2461; found, 1243.2449.

Elemental analysis: Calc. for C_58_H_40_N_8_Pd_2_: C, 68.63%; H, 4.32%; N, 8.89% found: C, 68.50%; H, 3.98%; N, 8.84%.

# 3. X-ray Crystallography

**Table S1.** Crystal data and X-ray experimental details of complex **2** and **3**.

|  | R2 | **2** | **3** |
| --- | --- | --- | --- |
| CCDC number | 2366852 | 2310045 | 2284482 |
| Empirical formula | C_51_H_38_Cl_2_N_4_O_4_Pd_2_ | C_54_H_58_N_8_Pd_2_ | C_75_H_58_Cl_16_N_8_Pd_2_ |
| Formula weight | 1054.55 | 1031.88 | 1496.79 |
| Temperature/K | 170.00 | 213.00 | 100.00(10) |
| Crystal system | triclinic | monoclinic | monoclinic |
| Space group | P-1 | C2/m | P2_1_/n |
| a/Å | 13.6520(3) | 23.4766(18) | 13.8756(3) |
| b/Å | 14.3709(3) | 18.8083(15) | 17.5074(3) |
| c/Å | 23.6617(4) | 13.2235(9) | 27.4383(6) |
| α/° | 78.0300(10) | 90 | 90 |
| β/° | 81.5520(10) | 118.235(4) | 102.547(2) |
| γ/° | 79.1150(10) | 90 | 90 |
| Volume/Å^3^ | 4431.44(16) | 5144.2(7) | 6506.3(2) |
| Z | 4 | 4 | 4 |
| ρ_calc_g/cm^3^ | 1.581 | 1.332 | 1.528 |
| μ/mm^‑1^ | 5.452 | 4.037 | 0.851 |
| F(000) | 2120.0 | 2120.0 | 3032.0 |
| Crystal size/mm^3^ | 0.17 × 0.17 × 0.15 | 0.07 × 0.07 × 0.05 | 0.26 × 0.21 × 0.19 |
| Radiation | GaKα (λ = 1.34139) | GaKα (λ = 1.34139) | Mo Kα (λ = 0.71073) |
| 2θ range for data collection/° | 5.544 to 109.996 | 7.242 to 110.134 | 3.042 to 58.74 |
| Index ranges | -16 ≤ h ≤ 16, -17 ≤ k ≤ 17, -28 ≤ l ≤ 27 | -28 ≤ h ≤ 28, -22 ≤ k ≤ 22, -16 ≤ l ≤ 12 | -16 ≤ h ≤ 14, -23 ≤ k ≤ 23, -34 ≤ l ≤ 33 |
| Reflections collected | 78794 | 21999 | 45352 |
| Independent reflections | 16818 [Rint = 0.0730, Rsigma = 0.0557] | 5030 [Rint = 0.1009, Rsigma = 0.0798] | 14167 [Rint = 0.0377, Rsigma = 0.0456] |
| Data/restraints/parameters | 16818/0/1139 | 5030/166/360 | 14167/2036/1155 |
| Goodness-of-fit on F^2^ | 1.047 | 0.996 | 1.090 |
| Final R indexes [I>=2σ (I)] | R_1_ = 0.0467, wR_2_ = 0.1190 | R_1_ = 0.0657, wR_2_ = 0.1715 | R_1_ = 0.0807, wR_2_ = 0.2066 |
| Final R indexes [all data] | R_1_ = 0.0632, wR_2_ = 0.1301 | R_1_ = 0.1035, wR_2_ = 0.2036 | R_1_ = 0.0985, wR_2_ = 0.2179 |
| Largest diff. peak/hole/e Å^-3^ | 1.40/-2.26 | 0.68/-1.16 | 2.06/-2.39 |

**
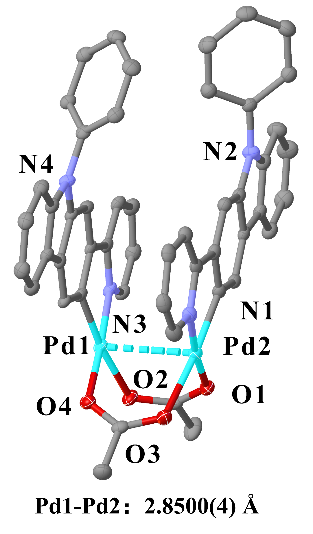
**

Figure S7. Olex2 representation of complex R2.

**
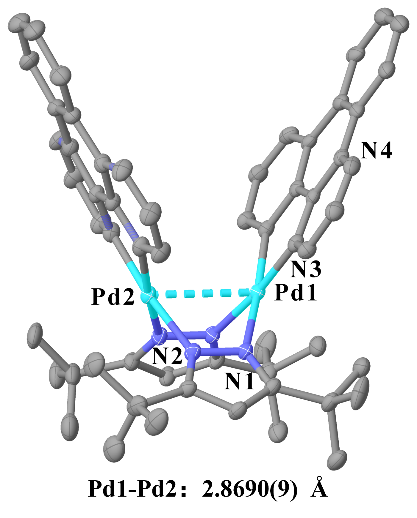
**

Figure S8. Olex2 representation of complex 2.


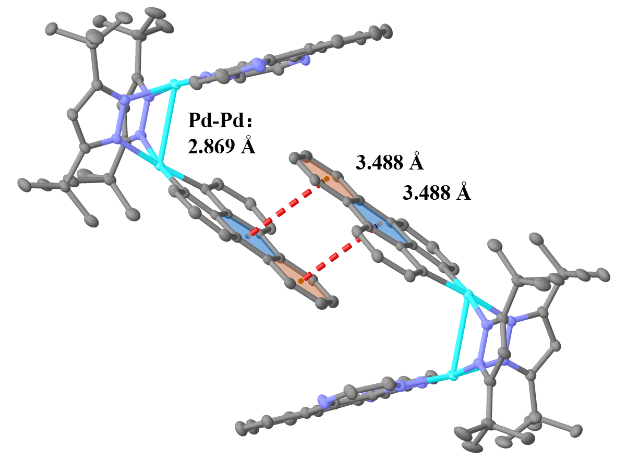


**Figure S9.** Intermolecular π-π interaction of complex **2.**

**Table S2**. Bond length of complex **2** bound to palladium atom [Å].

| **2** | bond length (Å) | **2** | bond length (Å) |
| --- | --- | --- | --- |
| Pd(1)-Pd(2) | 2.8690(9) | Pd(2)-N(2)1 | 2.090(5) |
| Pd(1)-N(1)1 | 2.083(5) | Pd(2)-N(2) | 2.090(5) |
| Pd(1)-N(1) | 2.084(6) | Pd(2)-C(26) | 2.028(6) |
| Pd(1)-N(3)1 | 2.009(6) | Pd(2)-C(26)1 | 2.028(6) |
| Pd(1)-N(3) | 2.009(6) | Pd(2)-N(5A) | 2.028(6) |
| Pd(1)-C(3A) | 2.009(6) |  |  |


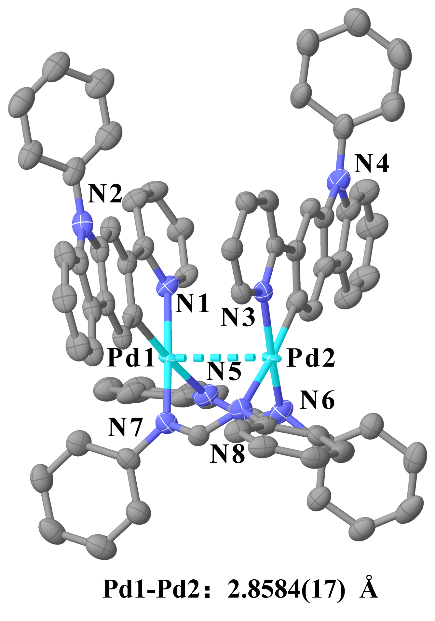


Figure S10. Olex2 representation of complex 3.


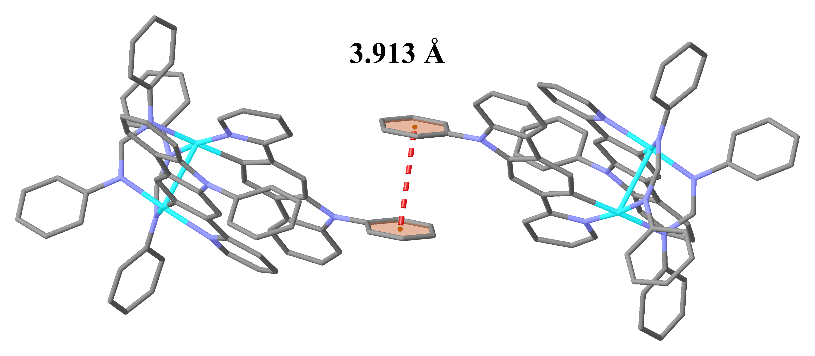


**Figure S11.** Intermolecular π-π interaction of complex **3**.


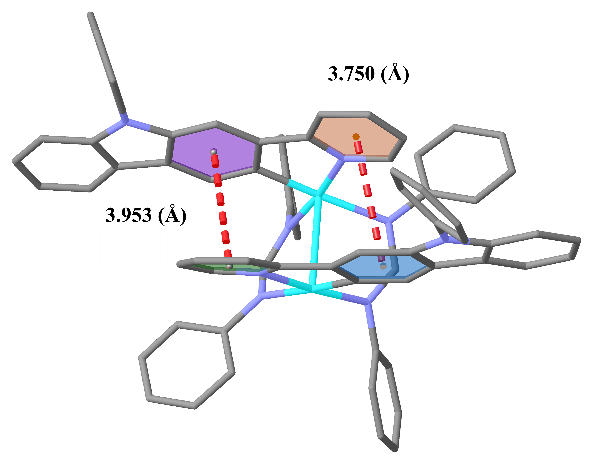


**Figure S12.** Intramolecular π-π interaction of complex **3**.


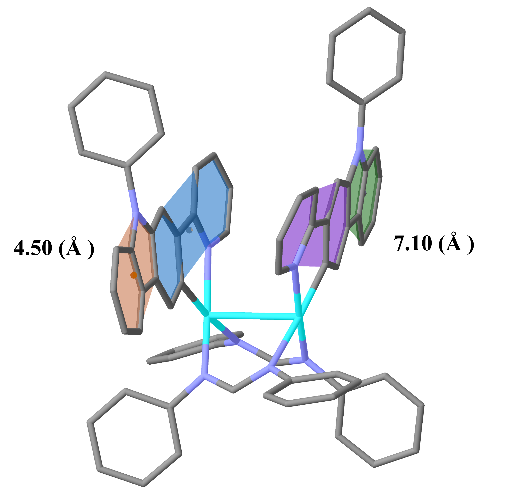


**Figure S13.** Dihedral angles between carbazole plane and pyridine plane of complex **3**.

**Table S3**. Bond length of complex **3** bound to palladium atom [Å].

| **3** | bond length (Å) | **3** | bond length (Å) |
| --- | --- | --- | --- |
| Pd(1)-Pd(2) | 2.8584(17) | Pd(2)-N(3) | 2.124(7) |
| Pd(1)-N(1) | 2.072(6) | Pd(2)-N(6) | 1.951(7) |
| Pd(1)-N(5) | 2.084(6) | Pd(2)-N(8) | 2.112(6) |
| Pd(1)-N(7) | 2.003(6) | Pd(2)-C(30) | 1.997(8) |
| Pd(1)-C(7) | 2.034(7) |  |  |

# 4. Photophysical Properties


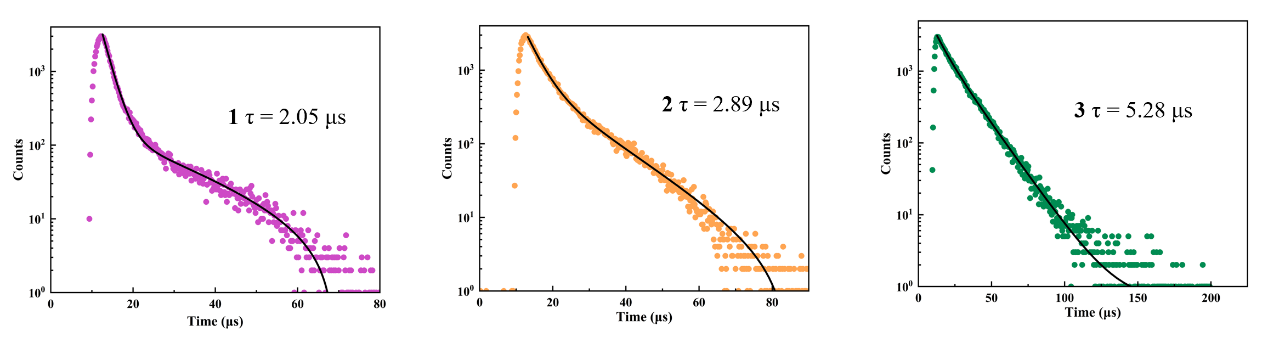


**Figure S14.** PL decay curves of complex **1**, **2** and **3** in solid state.


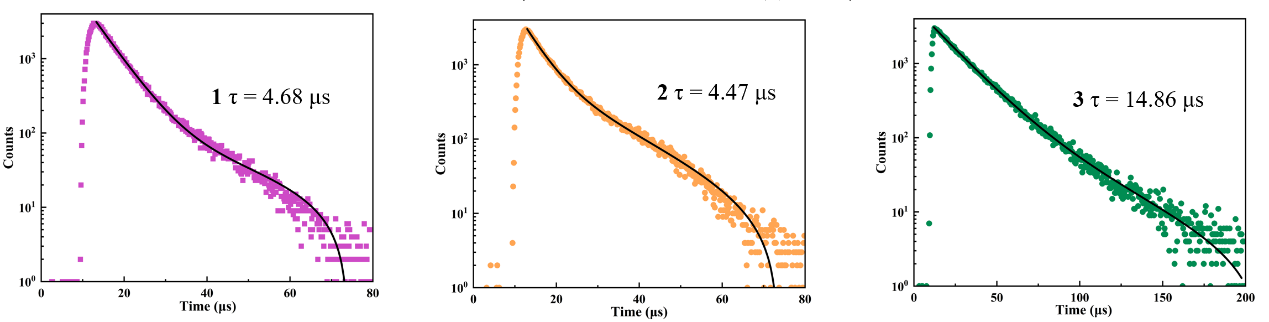


**Figure S15.** PL decay curves of complex **1**, **2** and **3** in 2 wt.% PMMA film state.


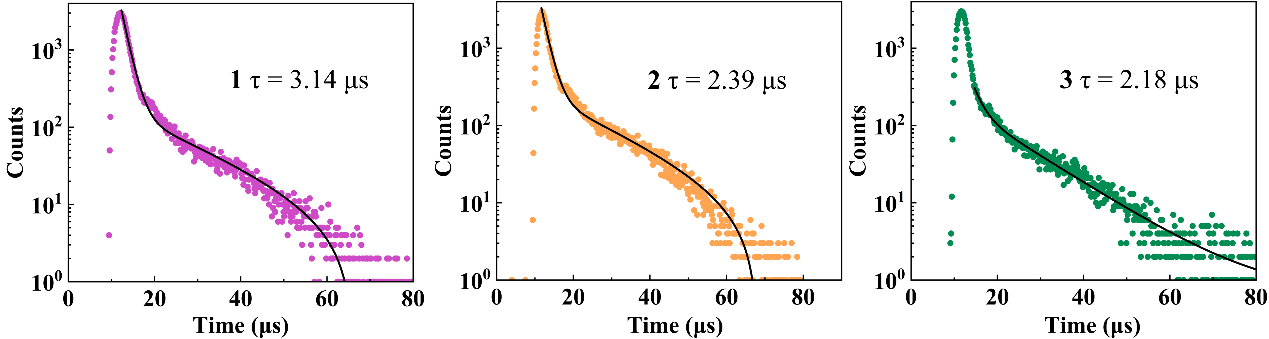


Figure S16. PL decay curves of complex 1, 2 and 3 in CH_2_Cl_2_ solution.


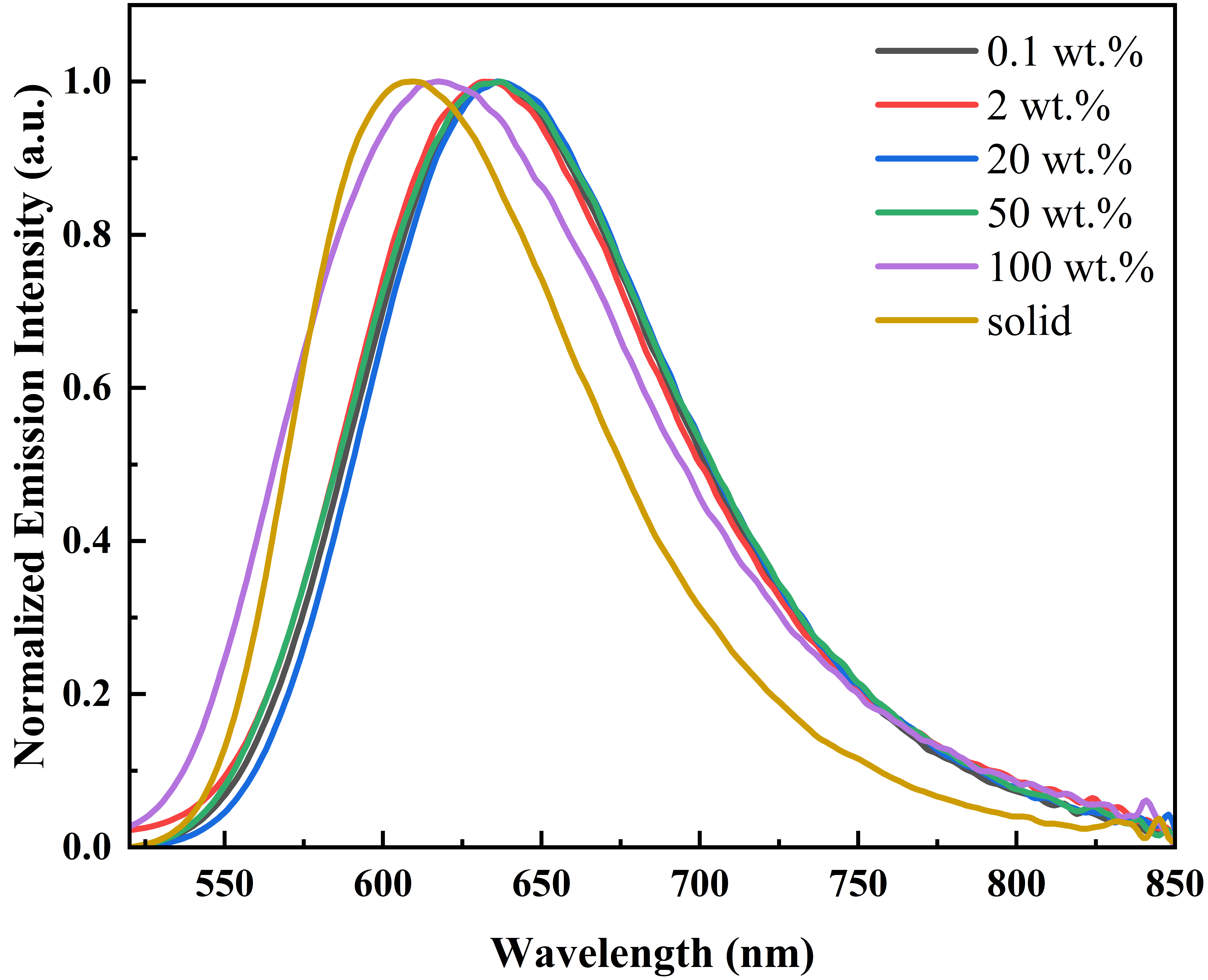


**Figure S17.** Emission spectra of complex **2** at different doping concentrations in polymethyl methacrylate (PMMA).


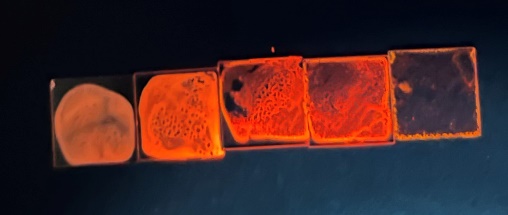


**Figure S18.** Physical picture of thin film of complex **2** in PMMA at different doping concentrations (from left to right, doping concentrations of 0.1 wt.%,2 wt.%,20 wt.%,50 wt.%, 100 wt.%).


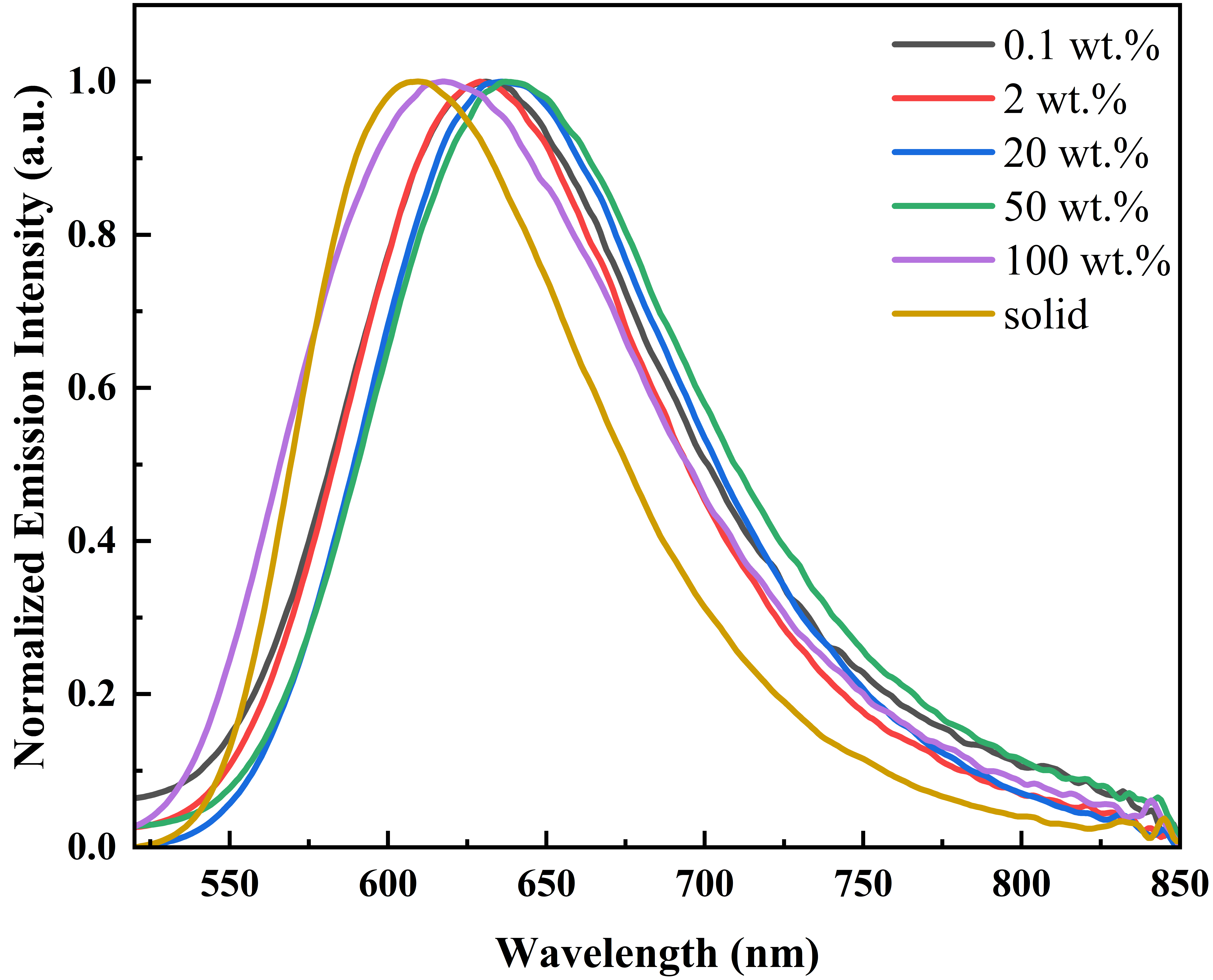


**Figure S19**. Emission spectra of complex **2** at different doping concentrations in polystyrene (PS).


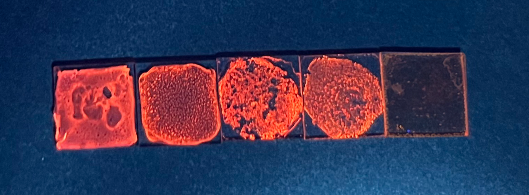


**Figure S20**. Physical picture of thin film of complex **2** in PS at different doping concentrations (from left to right, doping concentrations of 0.1 wt.%,2 wt.%,20 wt.%,50 wt.%, 100 wt.%).

# 5. Cyclic voltammetry data

**
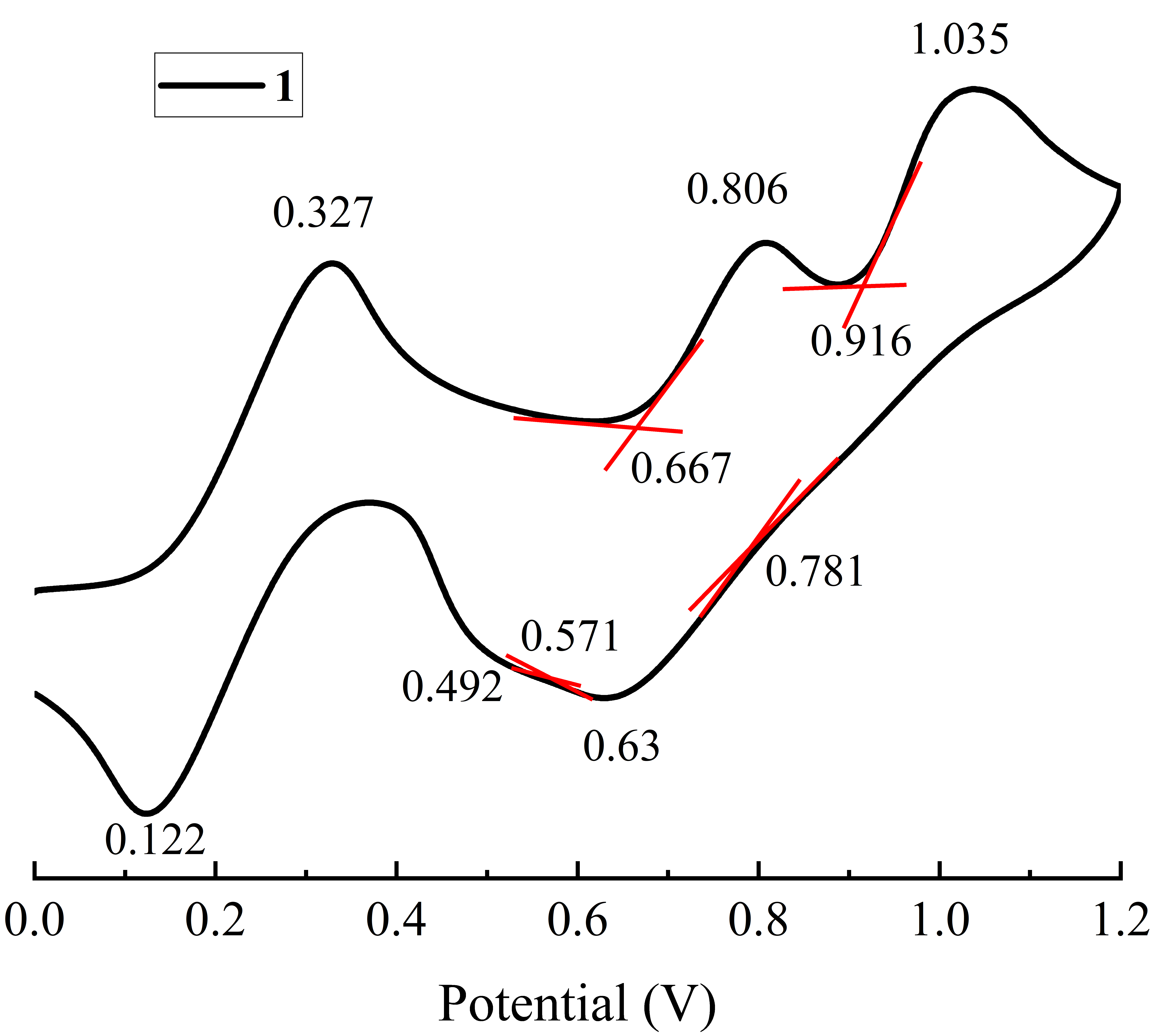
**

**Figure S21**. Cyclic voltammetry data of complex **1**.

**
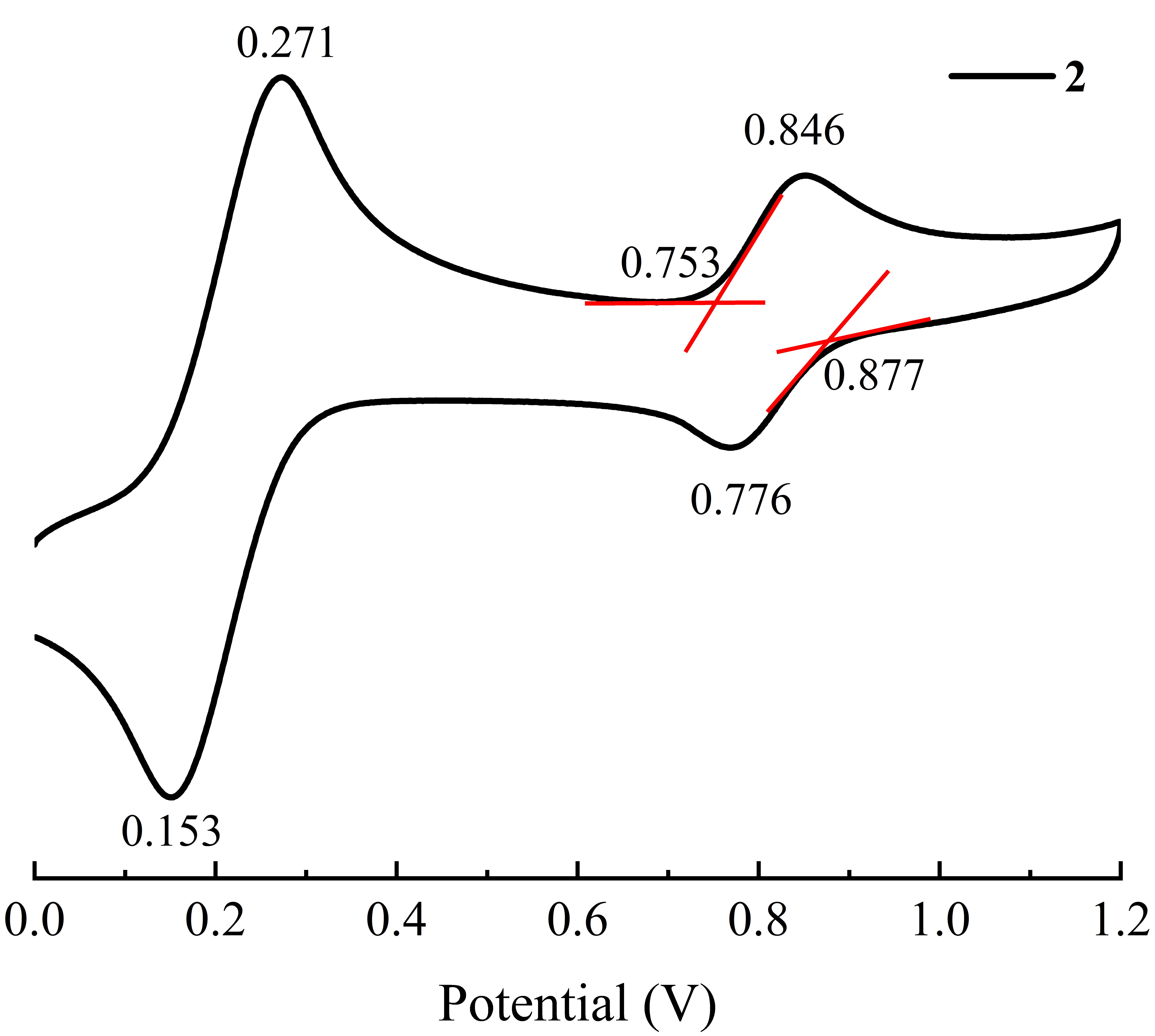
**

**Figure S22**. Cyclic voltammetry data of n complex **2**.

**
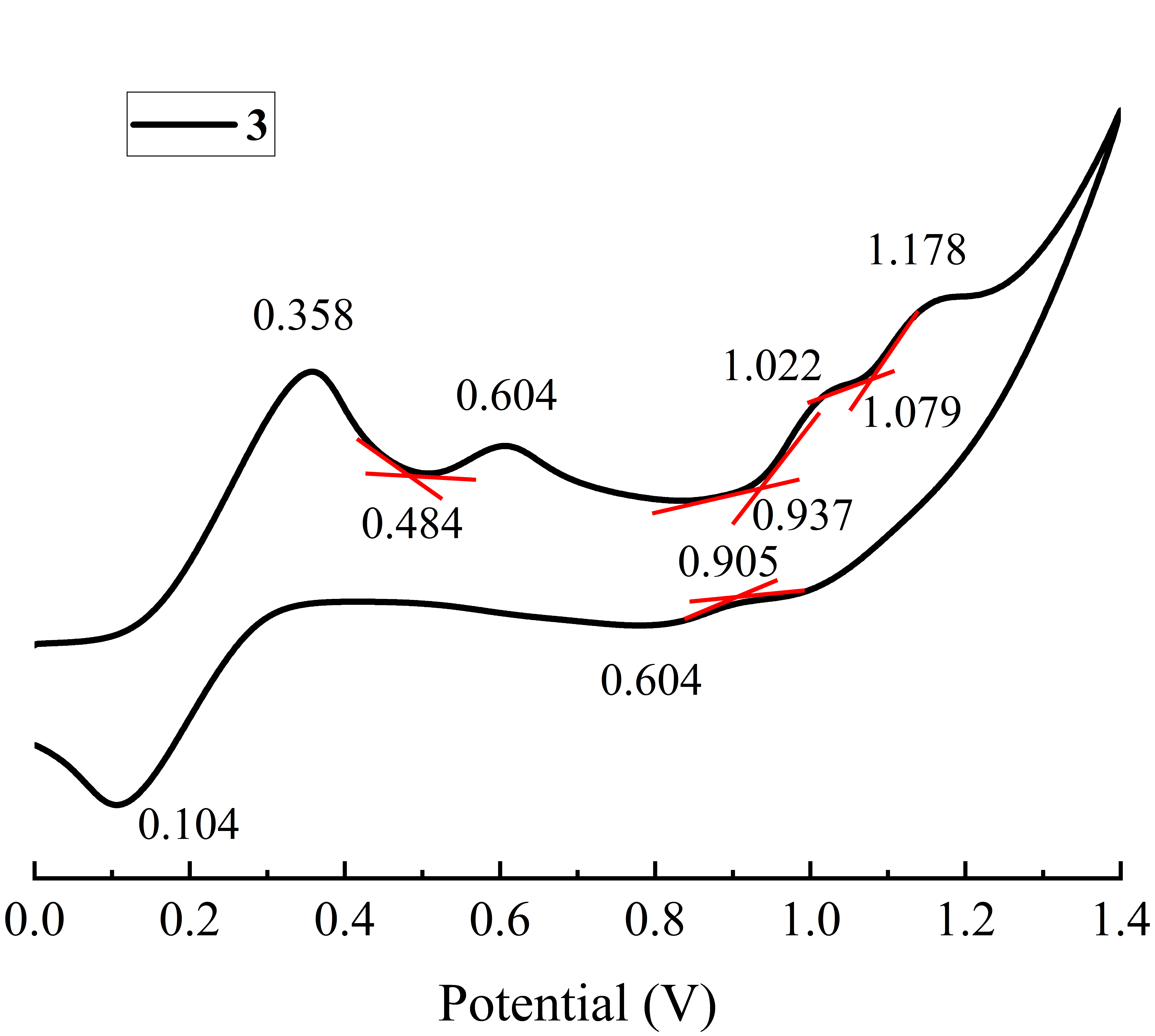
**

**Figure S23**. Cyclic voltammetry data of complex **3**.

# 7. OLEDs fabrications

**
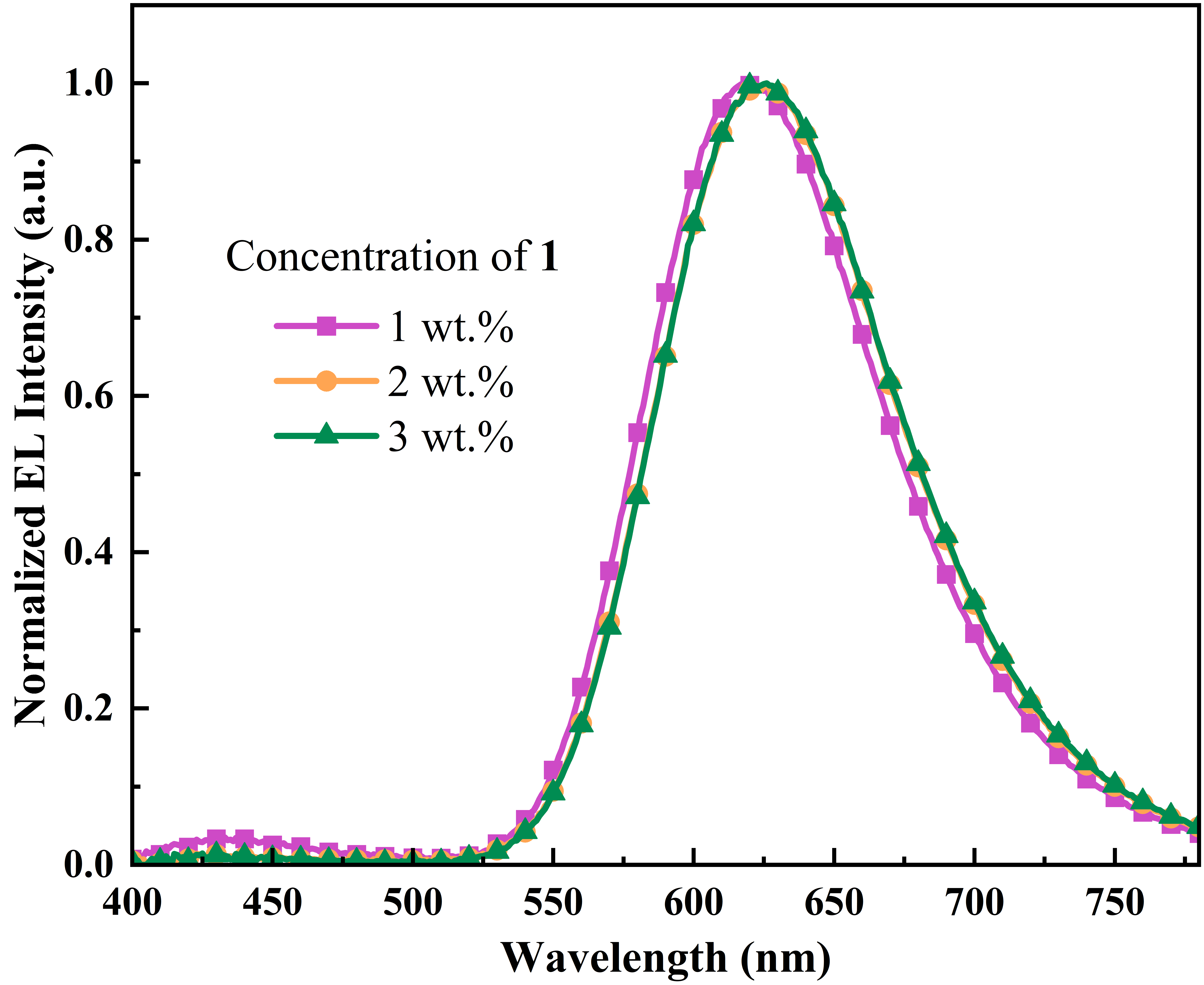
**

Figure S24. Normalized electroluminescence spectra of single-EML devices.

**
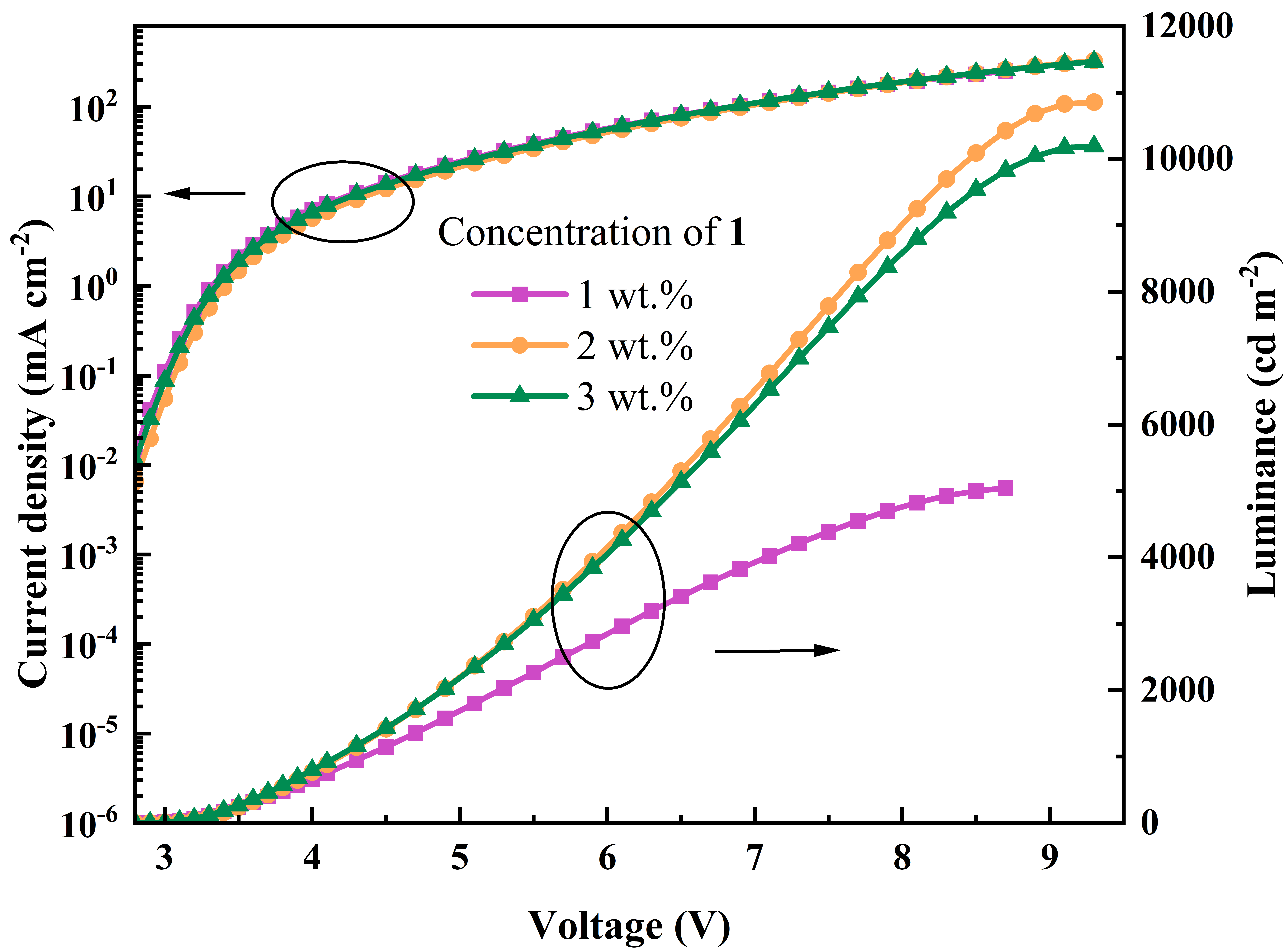
**

Figure S25. Current Density-Voltage-Luminance (J-V-L) characterization of single-EML devices.

**
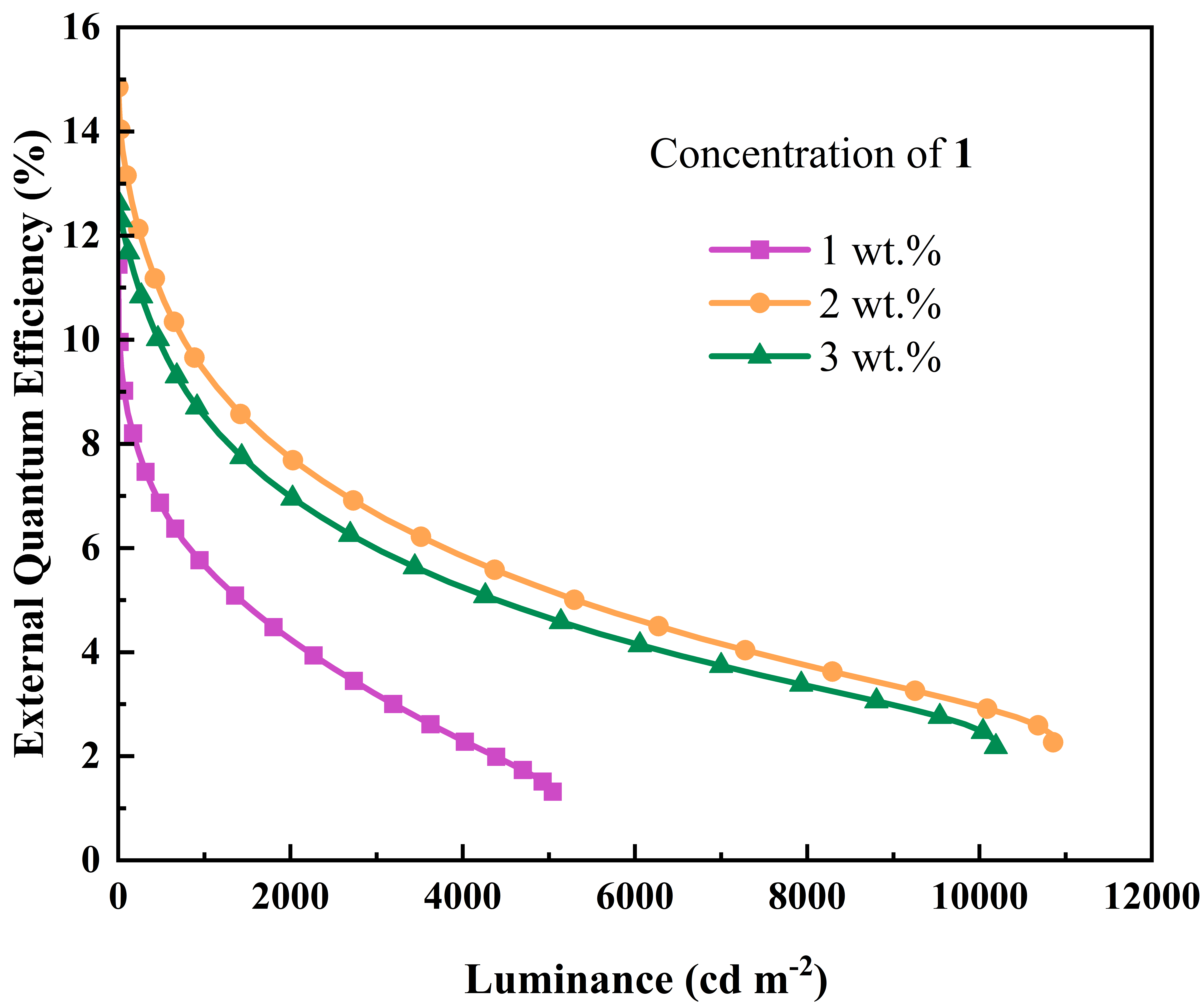
**

Figure S26. External quantum efficiency (EQE) characteristics of single-EML devices.

**Table S4**. Key properties of complex **1** for single-EML device at different doping concentrations.

| Device | L *^a^* (cd m^-2^) | η_c_ *^b^* (cd A^-1^) | η_p_*^c^* (lm W^-1^) | EQE *^d^* (%) | CIE_x, y_ *^e^* |
| --- | --- | --- | --- | --- | --- |
| 1 wt% | 5046 | 14.04 | 15.21 | 10.14 | (0.59, 0.38) |
| 2 wt% | 10856 | 18.09 | 19.60 | 14.85 | (0.61, 0.38) |
| 3 wt% | 10193 | 15.49 | 16.33 | 12.62 | (0.61, 0.38) |

*^a^* The data for maximum Luminance (L). *^b^* Maximum current efficiency (η_c_). *^c^* Maximum power efficiency (η_p_). *^d^* Maximum external quantum efficiency (EQE). *^e^* Commission Internationale de l’Eclairage coordinates (CIE_x, y_) at 10 mA cm^-2^.


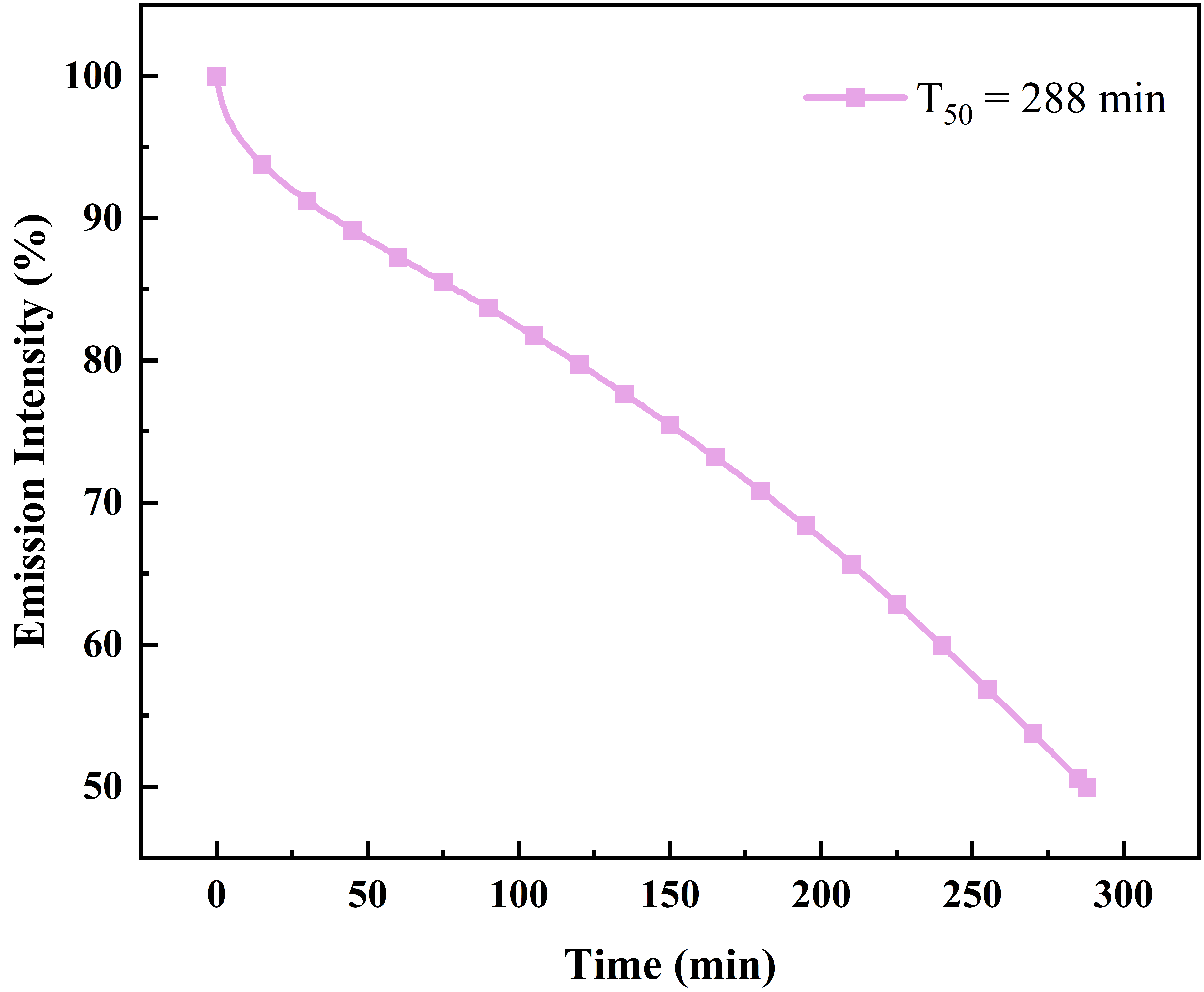


**Figure S27**. Operation lifetime of devices at an initial luminance of 1162 cd m^-2^.

In order to study the stability of the device, the operation lifetime of a double-emitting layers device with a doping concentration of 3 wt.% was measured at an initial luminance of 1162 cd m^-2^. As shown in Figure S27, the operating lifetime of the device is 288 minutes, which proves the stability of the device.

# 5. NMR and HRMS

**
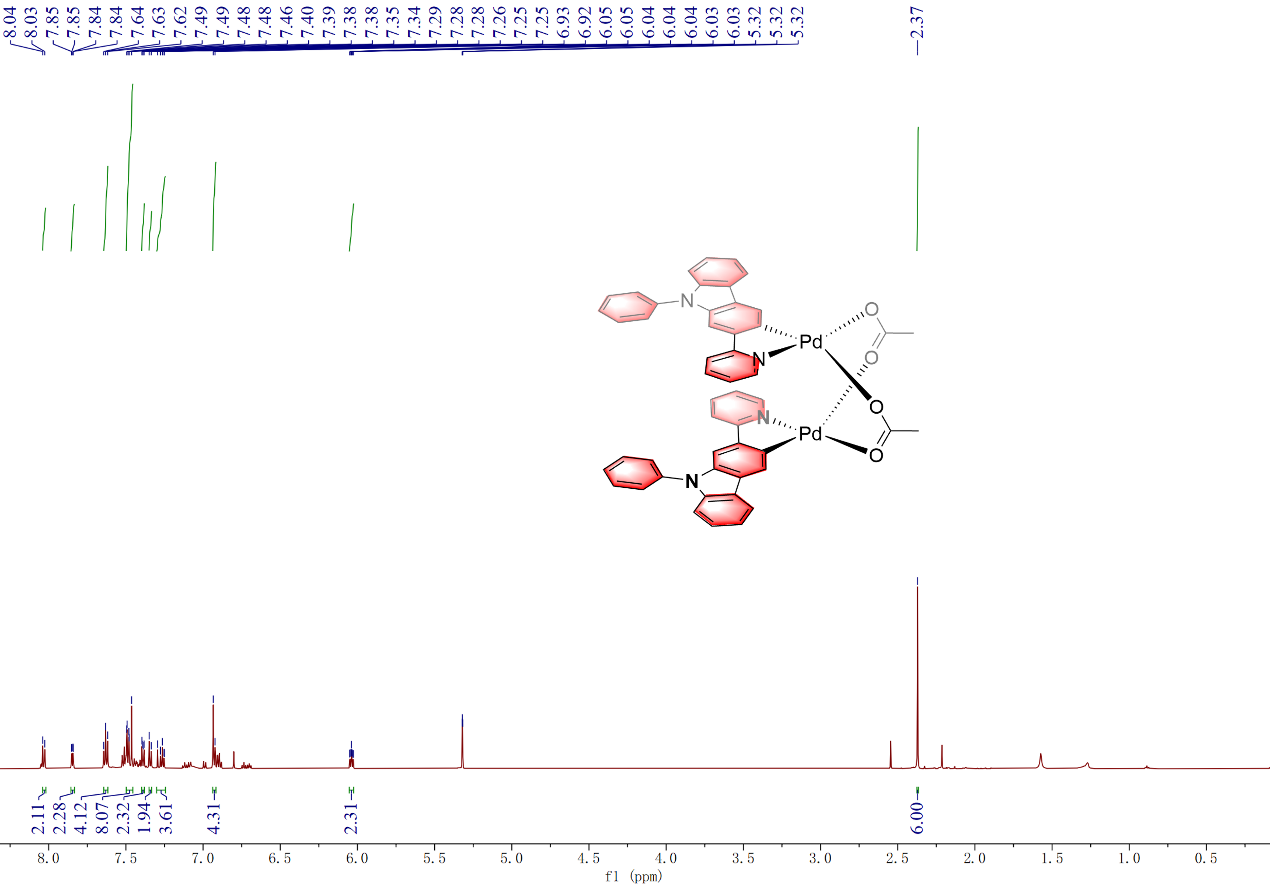
**

Figure S28. ^1^H NMR spectrum of complex R2 in CD_2_Cl_2._

**
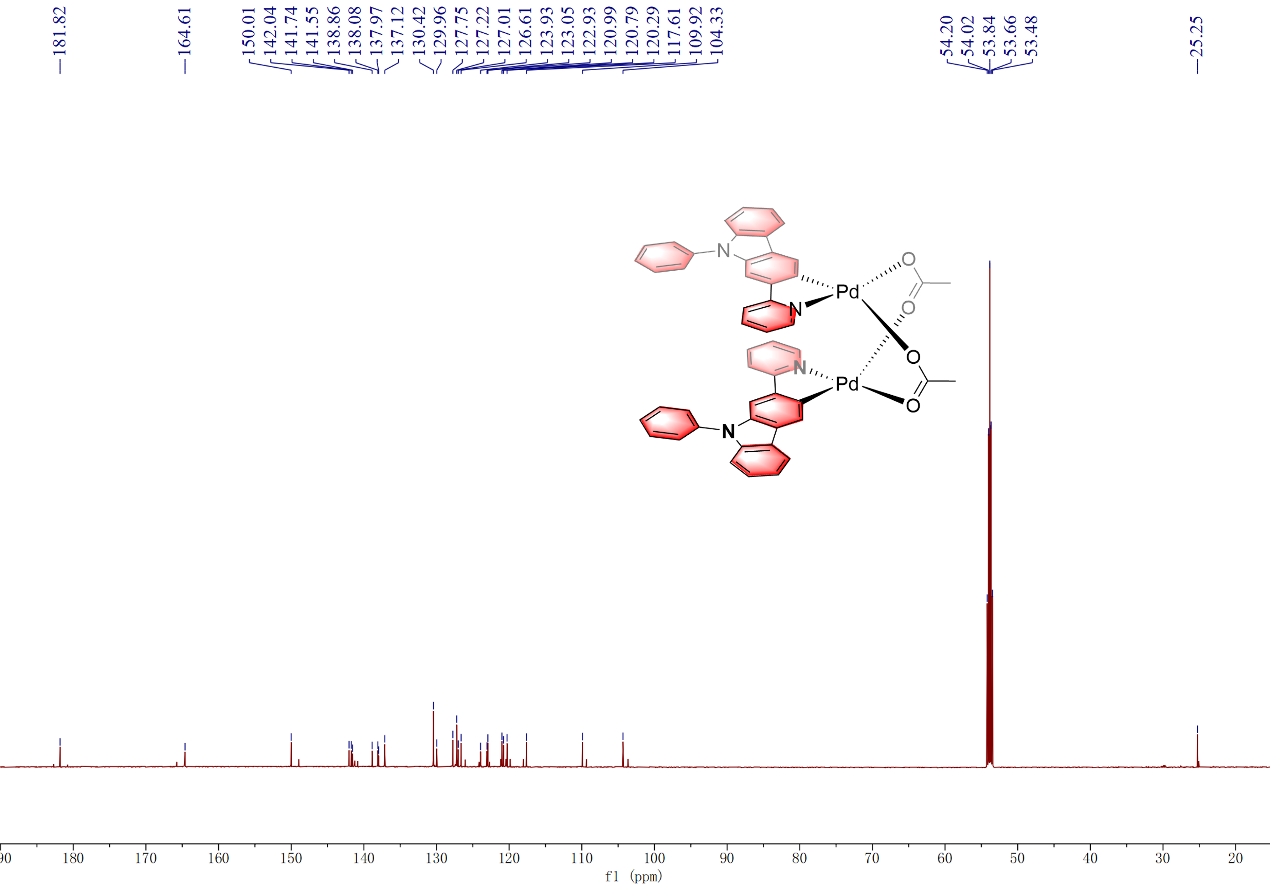
**

Figure S29. ^13^C NMR spectrum of complex R2 in CD_2_Cl_2._

**
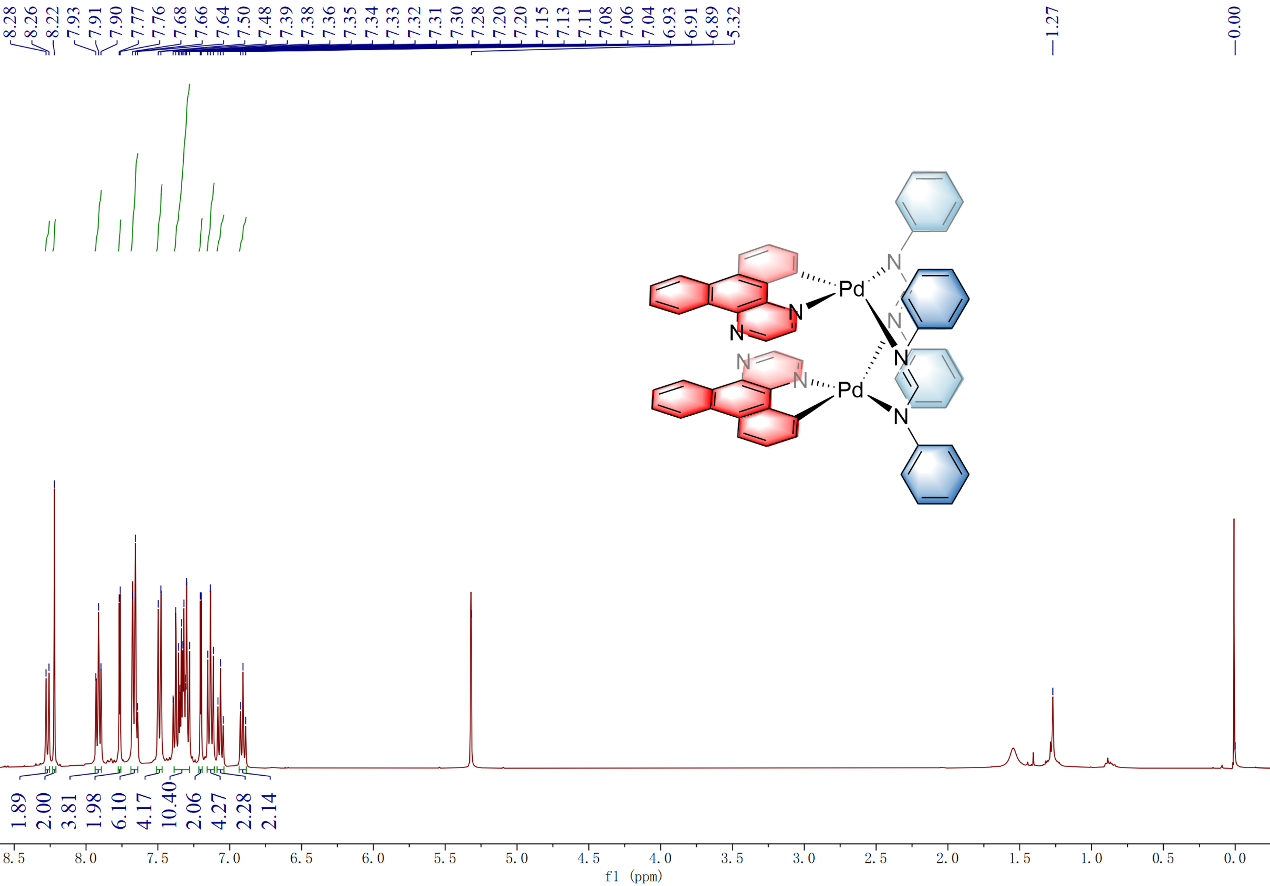
**

Figure S30. ^1^H NMR spectrum of complex 1 in CD_2_Cl_2._

**
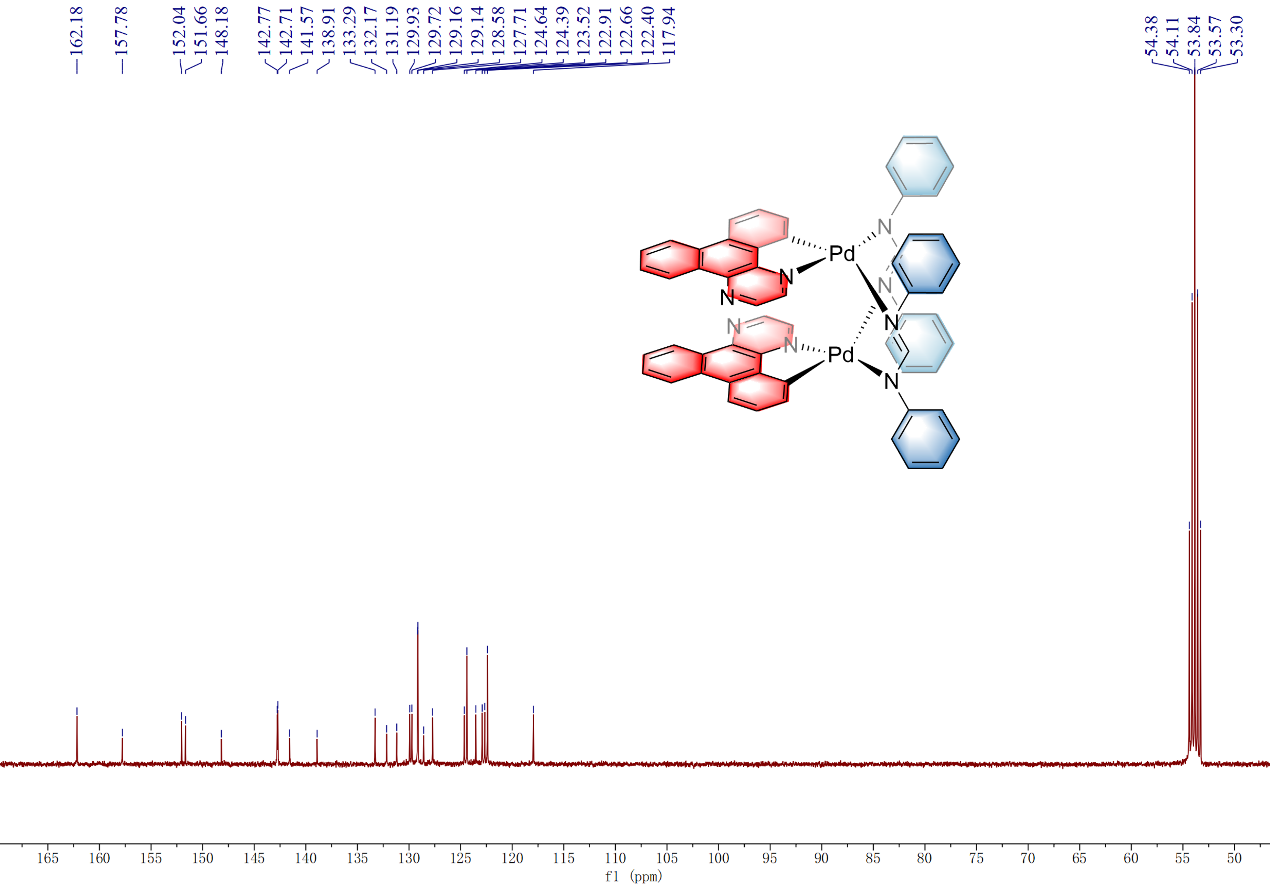
**

Figure S31.^13^C NMR spectrum of complex 1 in CD_2_Cl_2._

**
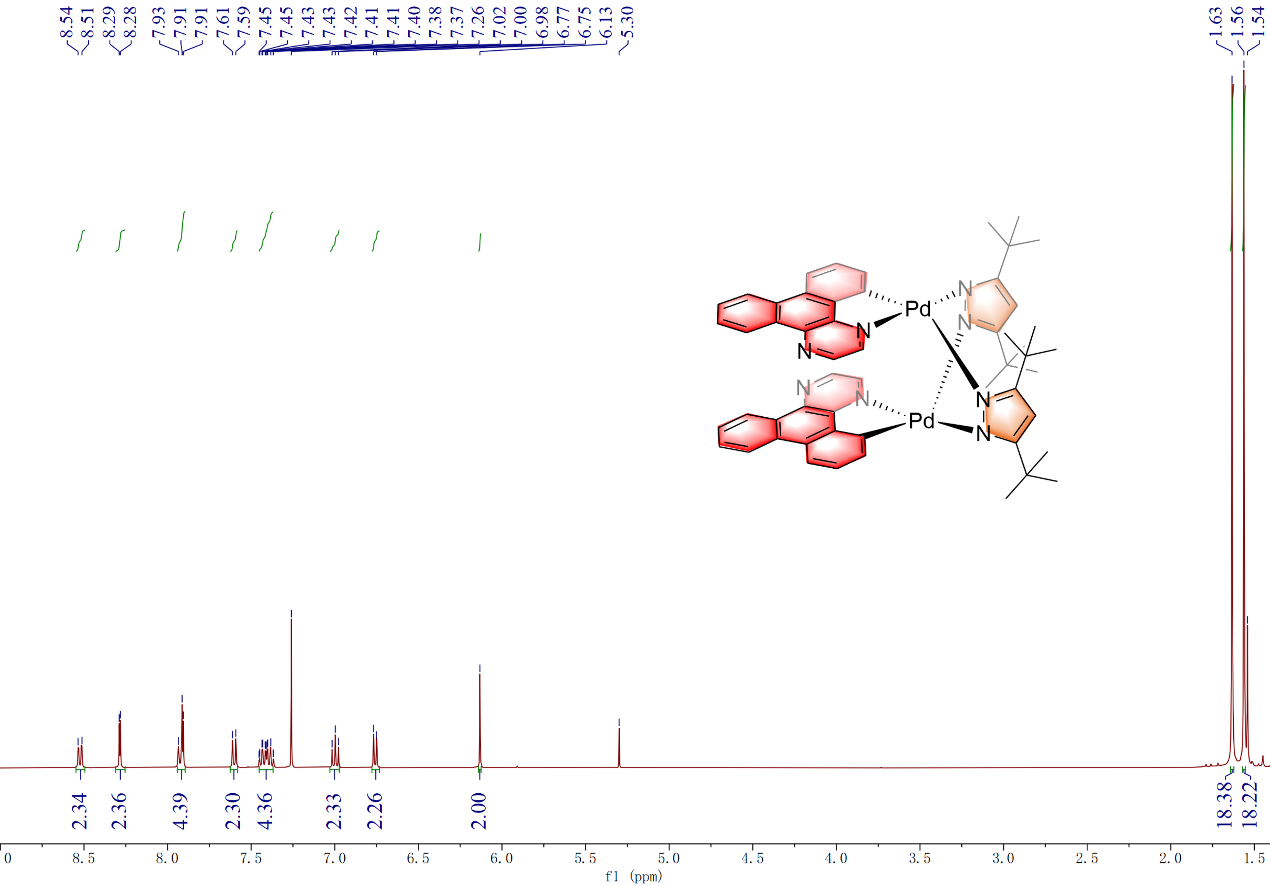
**

Figure S32. ^1^H NMR spectrum of complex 2 in CDCl_3._

**
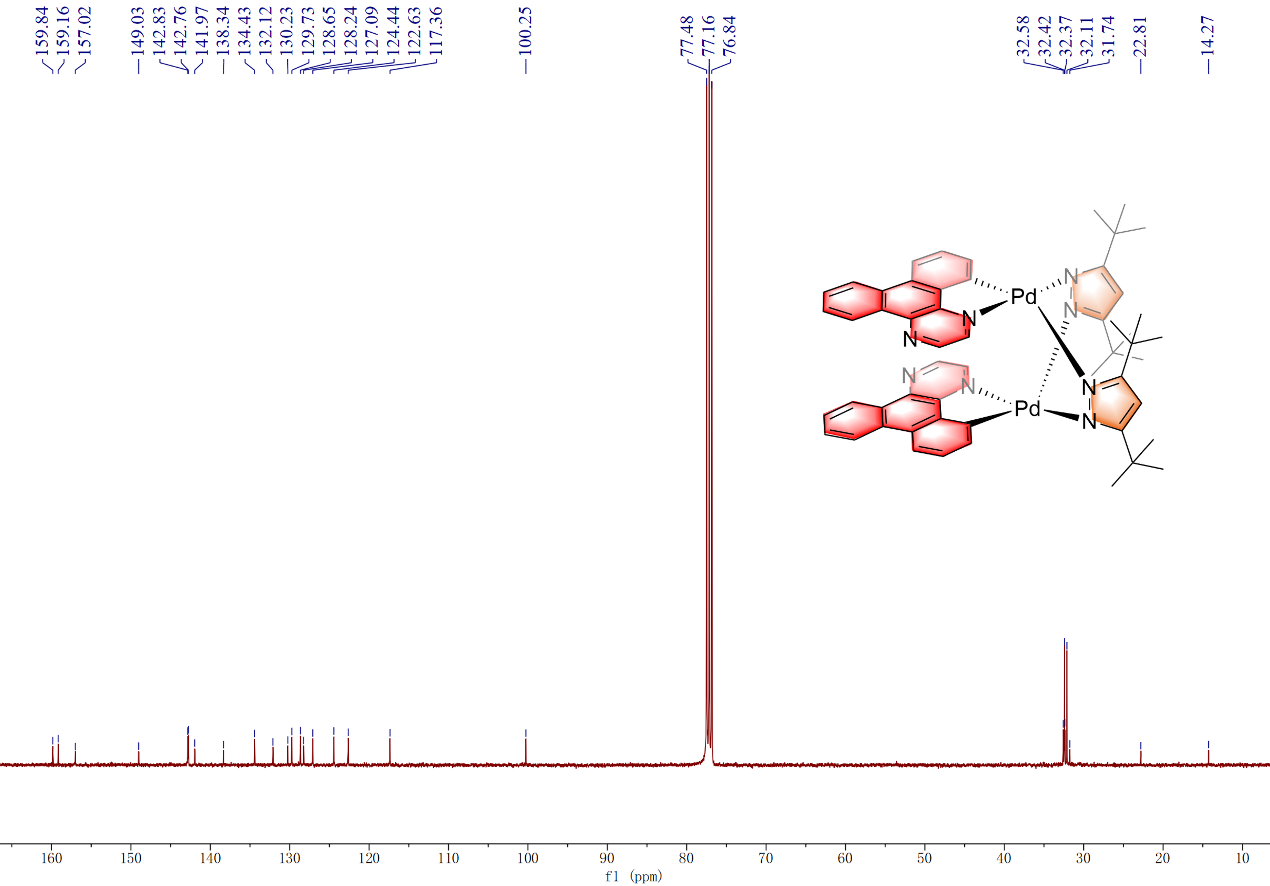
**

Figure S33. ^13^C NMR spectrum of complex 2 in CDCl_3._

**
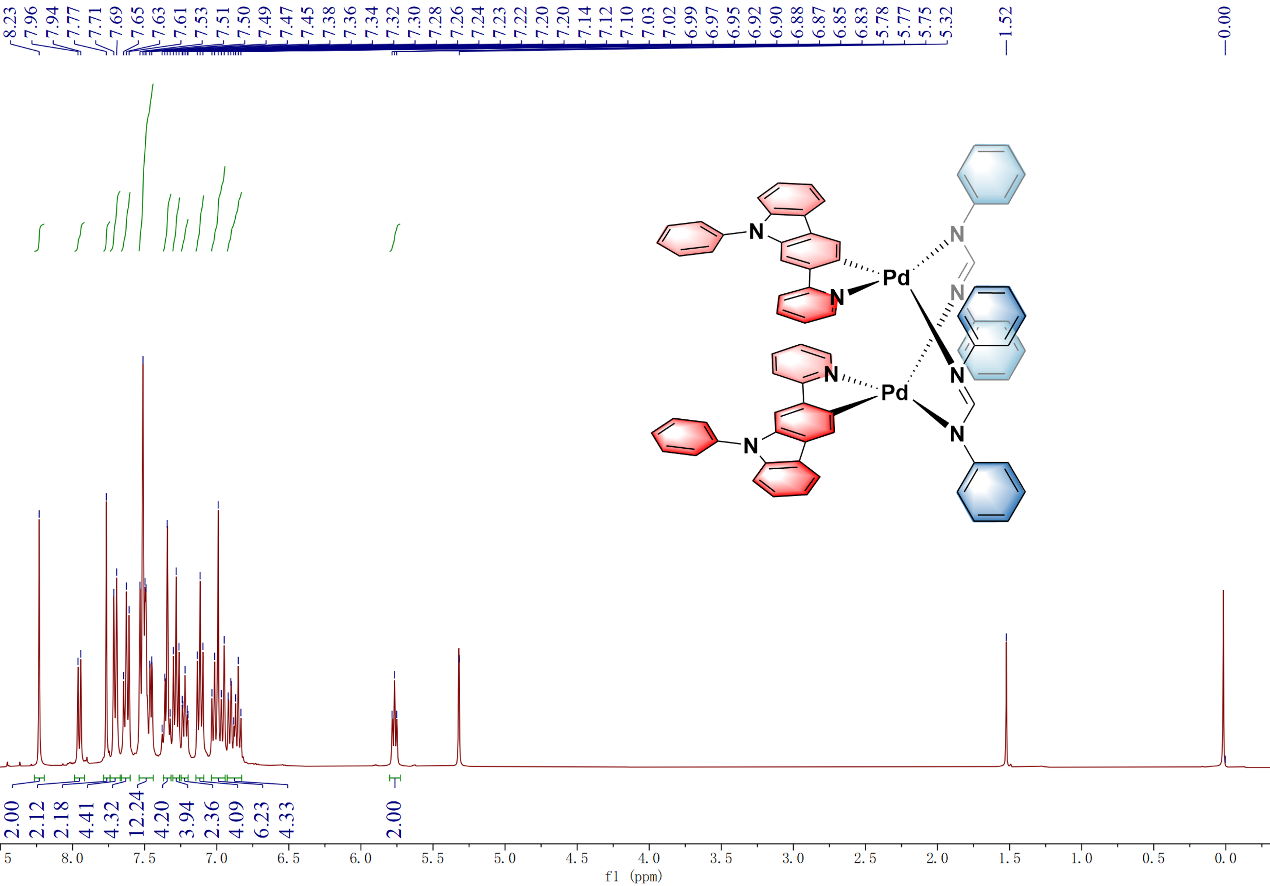
**

Figure S34. ^1^H NMR spectrum of complex 3 in CD_2_Cl_2._

**
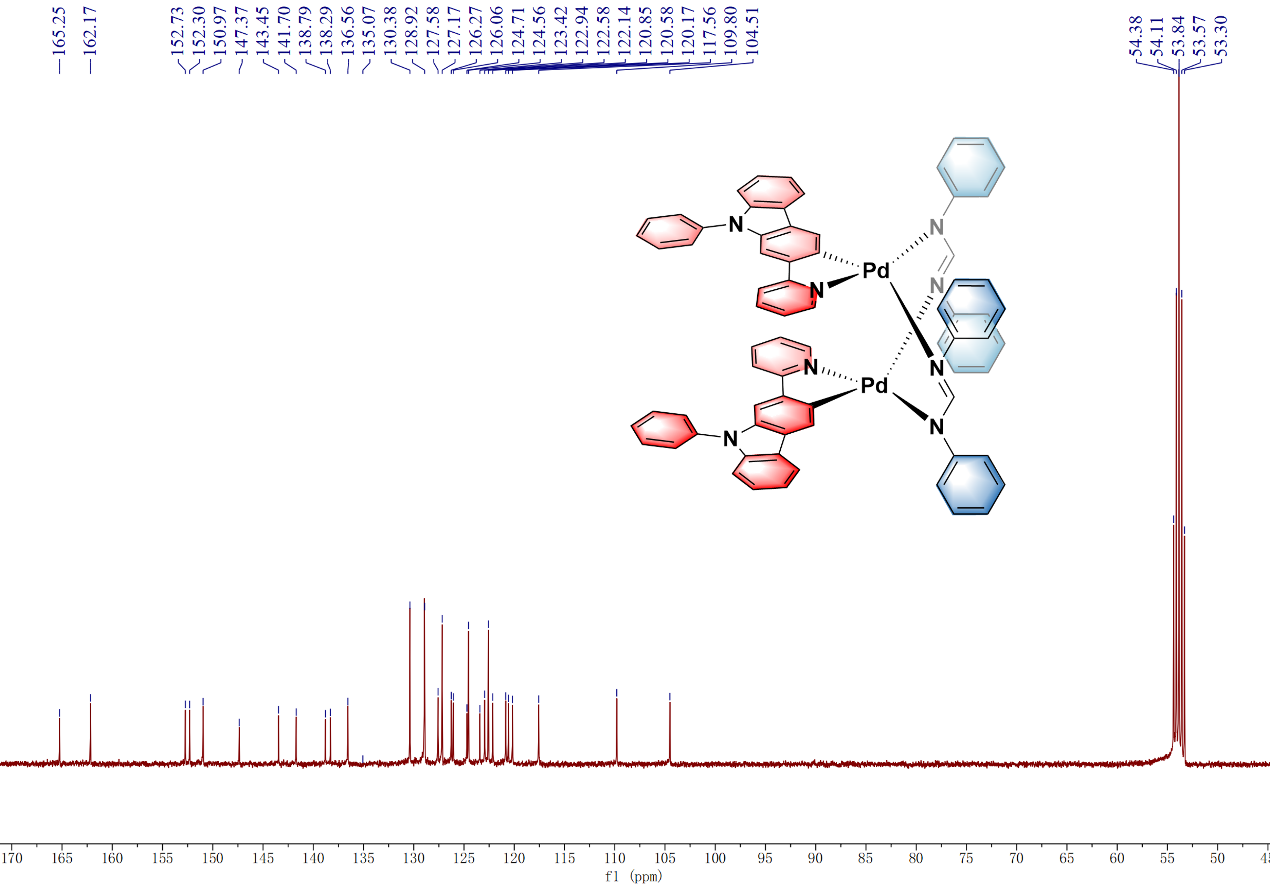
**

Figure S35.^13^C NMR spectrum of complex 3 in CD_2_Cl_2._


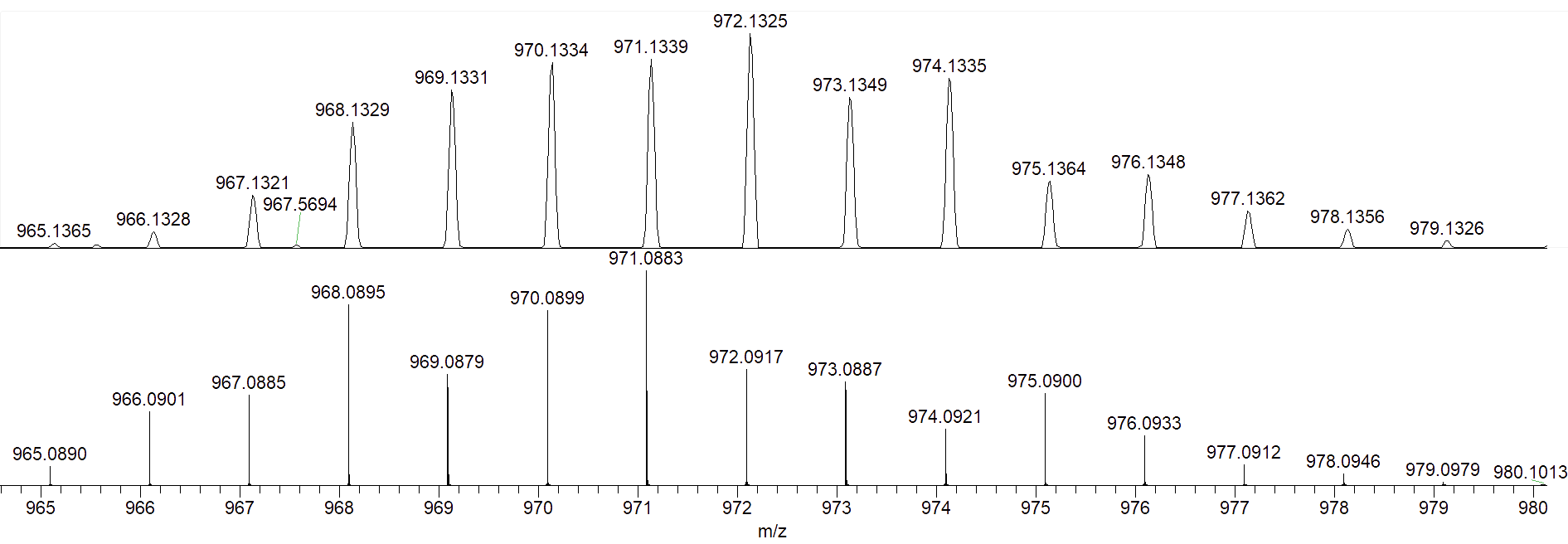


Figure S36. HRMS spectra for complex R2.

**
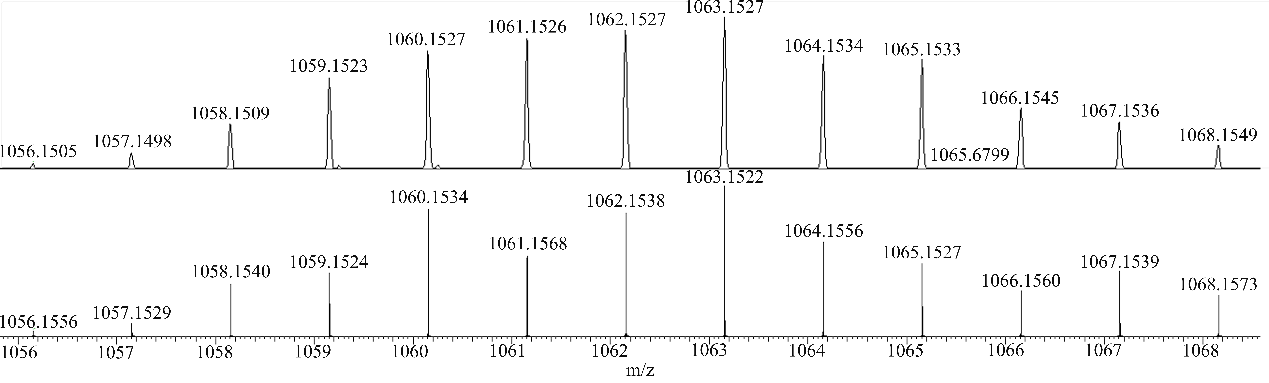
**

**Figure S37**. HRMS spectra for complex **1**.


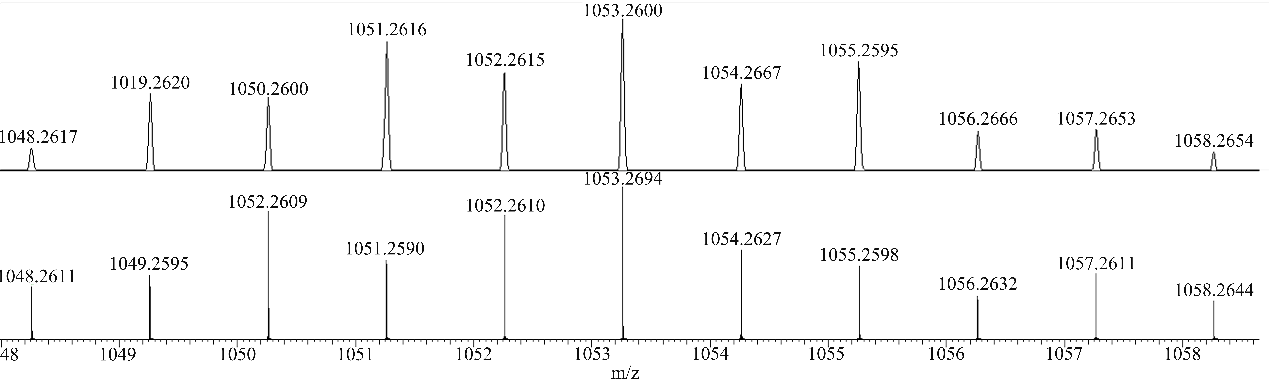


Figure S38. HRMS spectra for complex 2.


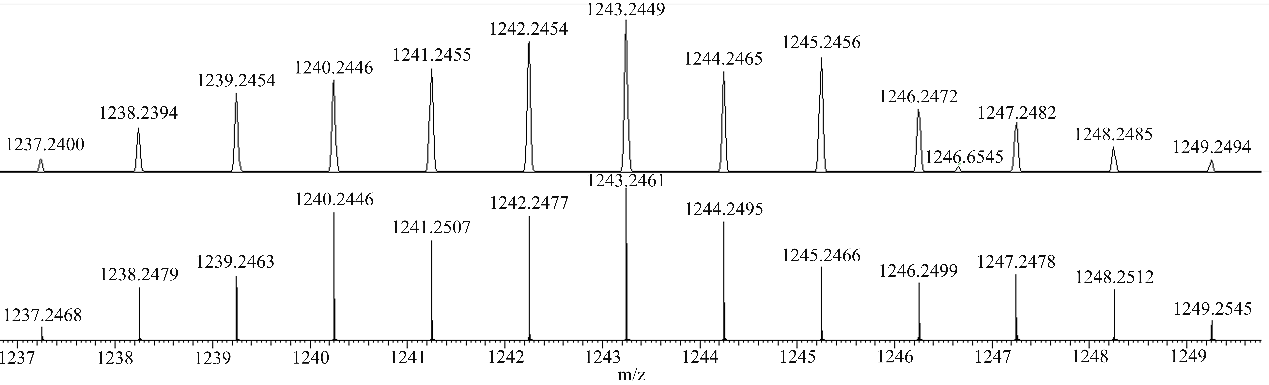


Figure S39. HRMS spectra for complex 3.

# 8. References

1. J. K. Kwon, J. H. Cho, Y.-S. Ryu, S. H. Oh, E. K. Yum, *Tetrahedron.* **2011**, 67, 4820-4825.
2. J. L. Van Wyk, B. Omondi, D. Appavoo, I. A. Guzei, J. Darkwa, *J. Chem. Res.* **2012**, 36, 474-477.
3. F. Zhou, D. S. Wang, X. Guan and T. G. Driver, *Angew. Chem. Int. Ed.* **2017**, 56, 4530-4534.
